# Limbic Alzheimer’s co-pathology in multiple system atrophy is associated with cognitive impairment and diagnostic inaccuracy

**DOI:** 10.1007/s00401-025-02940-0

**Published:** 2025-09-18

**Authors:** Janna van Wetering, Natasja A. C. Deshayes, Joëlle Boone, Inês Rodrigues Fernandes, Dagmar H. Hepp, Henk W. Berendse, Laura E. Jonkman, Annemieke J. M. Rozemuller, Wilma D. J. van de Berg

**Affiliations:** 1 Section Clinical Neuroanatomy and Biobanking, Department of Anatomy and Neurosciences, Amsterdam UMC, Vrije University, De Boelelaan 1117, 1081 HV Amsterdam, The Netherlands; 2 Amsterdam Neuroscience, Neurodegeneration Program, Amsterdam UMC, Amsterdam, The Netherlands; 3 Department of Neurology, Leiden University Medical Center, Leiden, The Netherlands; 4 Department of Neurology, Amsterdam UMC, Vrije University Amsterdam, Amsterdam, The Netherlands; 5 Department of Pathology, Amsterdam UMC, Vrije University Amsterdam, Amsterdam, The Netherlands

**Keywords:** Multiple system atrophy, Co-pathology, Amyloid-beta, Phosphorylated tau, Cognitive impairment, Diagnostic accuracy

## Abstract

The clinical heterogeneity of multiple system atrophy (MSA) may, along with the primary aggregation of alpha-synuclein (α-syn), partly be shaped by co-pathologies, such as amyloid-beta (Aβ), phosphorylated (p)-tau, and pTDP-43, though their relevance remains unclear. Here, we aimed to characterize the prevalence, morphology, regional patterns, and clinical relevance of co-pathologies in a well-characterized MSA autopsy cohort. Regional load (%area) and morphological characterization of α-syn (KM51), Aβ (6F/3D), p-tau (AT8), and pTDP-43 (pSer409) pathology were assessed in limbic regions of MSA (*n* = 70) donors from the Netherlands Brain Bank. APOE-ε4 genotyping and clinical parameters of the cohort were collected. Associations with clinical features were analyzed using ANCOVA and mixed linear models, adjusted for age and sex. Aβ, p-tau, and pTDP-43 pathology were detected in 31%, 91%, and 11% of all MSA cases, respectively, with the highest burdens in the entorhinal cortex and amygdala. Mixed MSA + AD cases had a higher age at death (75 ± 7 vs. 64 ± 7, *p* < 0.001), and more frequently APOE-ε4 alleles (*p* < 0.001) than pure MSA. Cognitive impairment (CDR scores) was associated with diffuse and compact Aβ plaques across all regions (r ≥ 0.24, *p* ≤ 0.015), p-tau pathology in CA1 and CA3 + 4 (r ≥ 0.32, *p* ≤ 0.020), and neuronal α-syn inclusions in the amygdala (r = 0.54, *p* < 0.001). No robust correlations were found between total α-syn burden and Aβ or p-tau. Misdiagnoses increased with co-pathology burden (Aβ: *p* = 0.040; p-tau: *p* = 0.020) and age at onset (80% in those with onset > 75 years). Our results demonstrate that limbic Aβ, p-tau, and neuronal α-syn pathologies occur in a substantial proportion of MSA donors and are independently associated with cognitive decline and diagnostic inaccuracy, particularly among those with older age at onset. By providing a systematic quantitative and morphological assessment of co-pathologies in limbic regions of MSA, our study advances beyond prior prevalence reports and highlights their direct clinical relevance. These findings highlight the need for refined diagnostic criteria and co-pathology-informed biomarker strategies for MSA.

**Supplementary Information:**

The online version contains supplementary material available at 10.1007/s00401-025-02940-0.

## Introduction

Multiple system atrophy (MSA) is a sporadic, and rapidly progressive neurodegenerative disorder clinically characterized by autonomic dysfunction, parkinsonism and/or cerebellar ataxia [1]. Patients are commonly wheelchair-bound within 5–8 years and are expected to survive between 6 and 10 years after disease onset only [2–4]. Based on the predominant motor phenotype, MSA is subclassified into a parkinsonian variant (MSA-P) or a cerebellar variant (MSA-C), reflecting the predominance of bradykinetic-rigid symptoms in MSA-P, and cerebellar symptoms in MSA-C [1, 5]. While cognitive impairment was long considered a diagnostic exclusion criterion for MSA [6], recent studies show that up to 37% of patients may develop cognitive dysfunction [7, 8]. Reflecting these insights, the 2022 Movement Disorder Society (MDS) diagnostic criteria no longer define cognitive impairment as an exclusion criterion, though dementia remains a red flag [1]. In spite of this, the antemortem diagnostic accuracy remains suboptimal: neuropathologically validated studies report clinical misdiagnoses in up to 38% of cases [7, 9–11], underscoring the diagnostic challenges in MSA.

Neuropathologically, MSA is characterized by widespread accumulation of misfolded alpha-synuclein (α-syn) in glial cytoplasmic inclusions (GCIs), accompanied by striatum- or cerebellum-predominant degeneration [12, 13], reflecting the clinical MSA-P or MSA-C phenotypes, respectively. Alongside the characteristic GCIs in oligodendrocytes, α-syn-positive astrocytes, neuronal cytoplasmic inclusions (NCIs), and possibly microglia have also been observed in MSA [14–16]. Mixed MSA with Lewy body pathology was reported in approximately 5% of the cases [17]. Moreover, emerging evidence implicates neuronal rather than glial α-syn accumulation as the main driver of neurodegeneration in MSA, challenging the traditional paradigm of primary oligodendroglial pathology in MSA [18].

In addition to α-syn pathology, co-pathologies—specifically amyloid-beta (Aβ), phosphorylated tau (p-tau) and phosphorylated transactive DNA-binding protein 43 (pTDP-43)—are increasingly recognized as modifiers of disease phenotype and progression in synucleinopathies and other neurodegenerative disorders [19, 20]. In MSA, the presence and clinical relevance of co-pathologies remain poorly defined [21]. Small-scale autopsy studies in MSA reported a frequency of p-tau pathology in up to 92%, Aβ in 14–38%, and pTDP-43 in 4–7% of cases, but their clinical correlates have not been systematically analyzed [22–24]. Sporadically, a mixed diagnosis of PSP and MSA has been reported (< 1%) [25–27]. A recent larger autopsy study, based on pathological staging, suggested a low burden of co-pathology in MSA with minimal clinical relevance [28]. Given the well-established link between Aβ pathology and APOE-ε4 genotype in the general population [29], we additionally assessed APOE-ε4 carrier status in the present study as part of the evaluation of Aβ-related disease mechanisms in MSA.

Although MSA-P and MSA-C are traditionally associated with degeneration of the striatum and cerebellum, respectively, this anatomical correspondence is not absolute. In practice, clinical and neuropathological phenotypes seem to show overlap, further complicating clinicopathological classification [30]. Furthermore, a higher burden of neuronal α-syn inclusions in limbic regions has been linked to cognitive symptoms in MSA [31]. In addition, cognitive symptoms were reported to be associated with higher Braak NFT stages and Thal Aβ phases in MSA [32]. These findings raise important questions about the contribution of co-pathologies to the clinical heterogeneity of MSA [22–24], particularly in relation to cognitive dysfunction [31, 32].

This study aims to (1) determine the prevalence, distribution, and morphological characteristics of p-tau, Aβ, and pTDP-43 co-pathologies in a neuropathologically confirmed MSA cohort (*n* = 70), and (2) evaluate their potential clinical relevance, particularly in relation to cognitive symptoms and diagnostic misclassification. Limbic structures are particularly vulnerable to co-pathologies across neurodegenerative diseases [33], likely due to their early-stage involvement in the proposed propagation of misfolded proteins, as described in the Braak staging of α-syn [34] and NFT [35], Thal phases of Aβ [36], and TDP-LATE-stages for pTDP-43 [37]. Accordingly, we chose the amygdala and hippocampus as a primary focus of the current study in order to explore shared pathological susceptibility across α-syn, p-tau, Aβ and pTDP-43 pathology in MSA. In addition to the regional burden of pathology, we further examined the morphological characteristics of protein aggregates, including inclusion type and cellular localization, to characterize the spectrum and limbic distribution of co-pathologies in MSA. Gaining a clearer picture of how different types and locations of co-existing brain pathologies vary in MSA may not only help to explain why symptoms differ between patients, but also lead to more accurate diagnoses. Unlike previous studies, which have primarily reported the pathological prevalence or general Braak [34, 35] and Thal [36] staging originally developed for PD or AD, the present study provides a systematic quantitative and morphological assessment of co-pathologies in limbic regions in MSA. By distinguishing between different plaque morphologies and cellular localizations of α-syn pathology, we aim to clarify their clinical relevance and contribute to diagnostic accuracy in MSA.

## Methods

### Study cohort

For this study, a total of 70 brain donors were included from the Netherlands Brain Bank (NBB; Amsterdam, the Netherlands; http://brainbank.nl). Written informed consent for brain autopsy, the use of brain tissue and the use of clinical information for research purposes was collected from all donors. All procedures of NBB were approved by the local ethical board of Amsterdam UMC (formerly known as VU Medical Center), Amsterdam. For donor characteristics, see Online Resource 1, part 1.

All MSA cases included by the NBB between 1995 and 2023 were screened for this study. Cases were included if they met the following neuropathological criteria for MSA: (1) degeneration of striatonigral and/or olivopontocerebellar structures, and (2) presence of α-syn-positive glial cytoplasmic inclusions [6]. MSA cases were categorized as pure MSA when Thal Aβ phase and Braak NFT stage were < 3 and as mixed MSA + AD when Thal Aβ stages or Braak NFT were ≥ 3. Only donors for whom clinical information on disease duration and predominant symptoms, and brain tissue samples were available, were included.

Clinical case records from all donors were systematically reviewed. Clinical features evaluated were: (1) sex; biological gender designated at birth, (2) age at onset; age at which the first symptom related to the neurological disorder was reported, (3) age at death, (4) disease duration; time in years between age at onset and age at death, (5) initial and last clinical diagnosis; as reported by treating physician, (6) estimated Hoehn and Yahr stage < 1 year prior to death; based on written clinical case records, (7) presence of parkinsonism + duration in years; bradykinesia with tremor and/or rigidity [1], (8) cerebellar syndrome + duration in years; (9) autonomic dysfunction + duration in years; specifically urinary dysfunction or orthostatic hypotension, (10) CDR score; as reported by the treating physician in the NBB questionnaire or based on the clinical case records < 1 year prior to death, (11) hallucinations; visual or acoustic hallucinations as mentioned in the clinical case records, (12) depressed mood + duration; as mentioned in the clinical case records, (13) REM behavioral sleep disorder (RBD); recorded as present if confirmed on polysomnography or if it was clinically suspected based on the behavioral description by the bed partner, (14) levodopa response + duration of medication use; as reported by patient and physician and subsequently ordinally categorized as never used/no/minimal/moderate/marked/significant. For case summaries, see Online Resource 1, part 2.

### Neuropathological assessment

Neuropathological diagnoses were performed by an experienced neuropathologist (A.J.M.R). Standardized assessment consisted of Braak α-syn stage [34], MSA α-syn stage [13], Braak NFT stage [35], Thal Aβ phases [32], TDP-LATE stage [37], cerebral amyloid angiopathy (CAA) type and stage [38], the presence of neuritic plaques assessed by Consortium to Establish a Registry for Alzheimer’s Disease (CERAD) score [39], and the presence of aging-related tau astrogliopathy (ARTAG) [40] and argyrophilic grain disease (AGD) [41] was reported. Formalin-fixed paraffin-embedded (FFPE) tissue blocks were obtained from the amygdala and middle hippocampus (HipMid) for detailed pathological characterization. Sections were cut at 6 μm thickness and mounted to superfrost® glass slides. Immunohistochemistry (IHC) was performed with antibodies against full-length α-syn (1/500, clone KM51, Monosan Xtra, The Netherlands), Aβ (1/500, clone 70 6F/3D, Dako, Denmark), p-tau (1/500, p-tau, clone AT8, Thermo Fisher Scientific, USA) and pTDP-43 (1/10.000, clone pSer409, Cosmo Bio, USA). Staining protocols for α-syn (KM51), Aβ (6F/3D) and p-tau (AT8) were previously published [42]. See Online Resource 1, part 3 for details of the pTDP-43 (pSer409) staining protocol.

Stained sections of α-syn, Aβ, and p-tau were quantitatively assessed after digitization with the Olympus VS200 slide scanner with a 20× objective. Regions of interest were manually delineated using QuPath software, version 0.5.0 [43], in which the amygdala was delineated as entire amygdaloid complex [44], and the hippocampus was divided into the following subregions: dentate gyrus (DG), cornu ammonis 1, 2 and 3 + 4 (CA1, CA2, CA3 + 4) and entorhinal cortex (EntC) [45] [examples in Online Resource 1, part 4]. In QuPath, an in-house developed script, previously published [42], quantified the percentage of total DAB-stained area of α-syn, Aβ and p-tau pathology, here referred to as %area [examples in Online Resource 1, part 5]. In addition, a semi-quantitative assessment was performed to classify inclusions based on morphology [examples in Online Resource 1, part 6]. For α-syn, absolute densities were calculated for glial cytoplasmic inclusions (GCIs) and neuronal inclusions, and the presence of α-syn neurites was scored on an ordinal scale (0–4). For Aβ, densities of diffuse, compact, and classic-cored plaques were measured. Similarly, for p-tau, absolute densities of pretangles and mature neurofibrillary tangles (NFTs) were assessed, along with ordinal scoring for thread severity (0–4). Pathological positivity was defined as follows: for α-syn, presence of GCIs, neuronal α-syn inclusions *or* ≥ mild α-syn neurites score; for Aβ, any presence of diffuse, compact or classic-cored plaques; for p-tau, presence of pretangles, mature NFTs or a ≥ mild p-tau threads score; and for pTDP-43, presence of cytoplasmic inclusions or threads. All slides were assessed by a primary rater (J.W.), with 20% independently re-evaluated by a second rater (N.D.) to assess inter-rater reliability. A two-way random-effects intraclass correlation coefficient (ICC2) and a mean absolute error (MAE) were calculated to quantify inter-rater reliability for pathology density ratings.

### Statistical analysis

IBM SPSS Statistics version 28.0.1.1 was used to compare demographics between disease groups using a Fisher’s exact test for categorical and a one-way ANOVA for continuous variables. A univariate ANCOVA adjusted for age at death and sex was performed to estimate differences in pathological burden, and corrected for multiple comparisons using the Bonferroni method. A linear mixed model (LMM) adjusted for age at death and sex was used to assess neuropathological and clinicopathological correlations across brain regions in R studio version 4.3.3, with standardized regression coefficients (r) and FDR correction for multiple comparisons [46]. Additionally, region-specific partial correlations between standardized pathological markers and clinical variables were computed in R studio using residuals from linear models adjusted for covariates, with significance set at FDR-corrected *p* < 0.05. Heatmaps and graphs were generated using GraphPad Prism version 10.2.0 (GraphPad Software, San Diego, California, USA) and Adobe Illustrator version 29.1 (Adobe Inc., San Jose, California, USA).

## Results

### Cohort demographics

The cohort consisted of 70 neuropathologically confirmed MSA donors, of which 43 (61%) were striatum-predominant, 18 (26%) were cerebellum-predominant, and 9 (13%) showed no clear predominant subtype. Clinical and neuropathological characteristics of striatum-predominant and cerebellum-predominant MSA subtypes are summarized in Online Resource 1, part 7.

Fourteen out of 70 donors (20%) were classified as mixed MSA + AD, of which two donors (#13 and #21, see Online Resource 1, part 1) exhibited a Thal Aβ phase of 4, with a corresponding Braak NFT stage of 1. Braak α-syn, MSA-SND, MSA-OPCA, and TDP-LATE stage were similar in pure MSA and mixed MSA + AD cases (Table 1). Age at onset (70 ± 7 vs. 58 ± 8) and age at death (75 ± 7 vs. 64 ± 7) were significantly higher in mixed MSA + AD than in pure MSA donors (*p* < 0.001) (Table 2). Estimated Hoehn and Yahr scores from the year before death were high (stage 4 or 5) and disease duration was similar (5 ± 2 vs. 6 ± 3) in both groups. Cognitive impairment, reflected by higher CDR scores, was more common in mixed MSA + AD (50% with CDR ≥ 1) than in pure MSA (7% with CDR ≥ 1; *p* < 0.001) cases. The prevalence of parkinsonism, cerebellar symptoms, autonomic dysfunction, hallucinations, depressed mood, RBD, and levodopa responsiveness did not differ between groups. Pathologically, most donors showed overlapping striatonigral degeneration and olivopontocerebellar changes. Only three individuals had isolated striatonigral degeneration without olivopontocerebellar involvement; these cases all had relatively rapid disease courses (3–4 years), and two deceased from respiratory complications.

**Table 1:** Neuropathological stages and APOE-ε4 genotype of study cohort

	Pure MSA	Mixed MSA + AD	*p* value^1^
*N*	56	14	
Pathological MSA subtype (%)			n.s
striatum-predominant N (%)	33 (59%)	10 (71%)	
cerebellum-predominant N (%)	15 (27%)	3 (21%)	
Mix (striatum = cerebellum) N (%)	8 (14%)	1 (7%)	
Braak α-syn stage [34] 0/1/2/3/4/5/6	13/0/1/12/8/12/10	2/0/0/5/3/4/0	n.s
MSA-SND stage [13] 0/1/2/3	0/8/14/34	0/2/2/10	n.s
MSA-OPCA stage [13] 0/1/2/3	2/21/15/18	1/5/6/2	n.s
Thal Aβ phase [36] 0/1/2/3/4/5	32/14/8/0/0/0	1/0/2/4/7/0	*p* < 0.001
Braak NFT stage [35] 0/1/2/3/4/5/6	13/30/13/0/0/0/0	0/4/0/6/3/1/0	*p* < 0.001
TDP-LATE^1^ stage [37] 0/1a/1b/2/3	46/3/1/1	9/2/0/1	n.s
CAA type [38] 0/1/2	41/4/8	3/3/8	*p* < 0.001
CAA stage [38] 0/1/2/3	41/9/2/1	3/7/3/1	*p* < 0.001
CERAD [39] 0/1/2/3	49/3/0/0	2/8/0/2	*p* < 0.001
ARTAG [40] 0/1/2	40/13/2	5/6/3	*p* = 0.013
AGD not mentioned/present	53/3	13/1	n.s
APOE-ε4 alleles Non-carrier/hetero-/homozygous	40/15/1	3/7/4	*p* < 0.001

N.s. = no significant difference between groups. SND = striatonigral degeneration; OPCA = olivopontocerebellar atrophy; TDP-LATE = Limbic-predominant age-related TDP-43 encephalopathy; CAA = cerebral amyloid angiopathy; CERAD = Consortium to Establish a Registry for Alzheimer Disease; ARTAG = aging-related tau astrogliopathy; AGD = argyrophilic grain disease; APOEε = apolipoprotein E. Groups were compared using a one-way ANOVA for continuous and a Fisher’s exact test for categorical variables

^a^TDP-LATE staging was missing for 7 donors

**Table 2:** Clinical features of study cohort

	Pure MSA	Mixed MSA + AD	*p* value^1^
*N*	56	14	
Sex M/F (%M)	21/36 (37%)	7/6 (54%)	n.s
Age at onset y mean ± SD (range)	58 ± 8 (41–81)	70 ± 7 (58–80)	*p* < 0.001
Age at death y mean ± SD (range)	64 ± 7 (48–84)	75 ± 7 (60–84)	*p* < 0.001
Disease duration y mean ± SD (range)	6 ± 3 (1—19)	5 ± 2 (1—9)	n.s
Final antemortem diagnosis MSA/not MSA (%matching postmortem)	49/7 (87%)	8/6 (57%)	*p* = 0.017
Final antemortem diagnosis specified			*p* = 0.027
MSA-P/MSA-C/MSA-unspecified/	23/10/16/	1/3/4/	
PD/PDD/PSP/AD/cerebellar atrophy	5/0/1/0/1	3/1/1/1/0	
Initial antemortem diagnosis specified			n.s
MSA-unspecified/PD/DLB/parkinsonism	22/25/1/5/	2/8/0/1/	
AD/cerebellar syndrome/MS/PTSD	0/2/0/1	1/1/1/0	
Cause of death euthanasia/other	24/32 (43%)	6/8 (43%)	n.s
Hoehn and Yahr estimated 0/1/2/3/4/5	0/0/0/0/13/43	0/0/0/0/7/7	n.s
CDR 0/0.5/1/2	34/18/4/0	4/3/4/3	*p* < 0.001
Parkinsonism yes/no (%yes)	50/6 (89%)	13/1 (93%)	n.s
Cerebellar symptoms yes/no (%yes)	44/8 (85%)	11/2 (85%)	n.s
Autonomic dysfunction yes/no (%yes)	53/1 (98%)	13/0 (100%)	n.s
Hallucinations yes/no (%yes)	10/10 (50%)	1/4 (20%)	n.s
Depressed mood yes/no (%yes)	30/13 (70%)	9/2 (82%)	n.s
RBD yes/no (%yes)	18/8 (70%)	4/1 (80%)	n.s
Levodopa response never used/none/minimal/moderate/marked/significant	8/8/17/5/9/0	2/3/2/2/2/0	n.s

Clinical characteristics were only reported if specifically stated as present or not present in clinical reports, not all information was available. N.s. = no significant difference between groups. CDR = clinical dementia rating score; RBD = REM behavioral sleep disorder. Groups were compared using a one-way ANOVA for continuous and a Fisher’s exact test for categorical variables

### Reduced diagnostic sensitivity in mixed MSA+AD donors and late−onset MSA

**Fig. 1:**
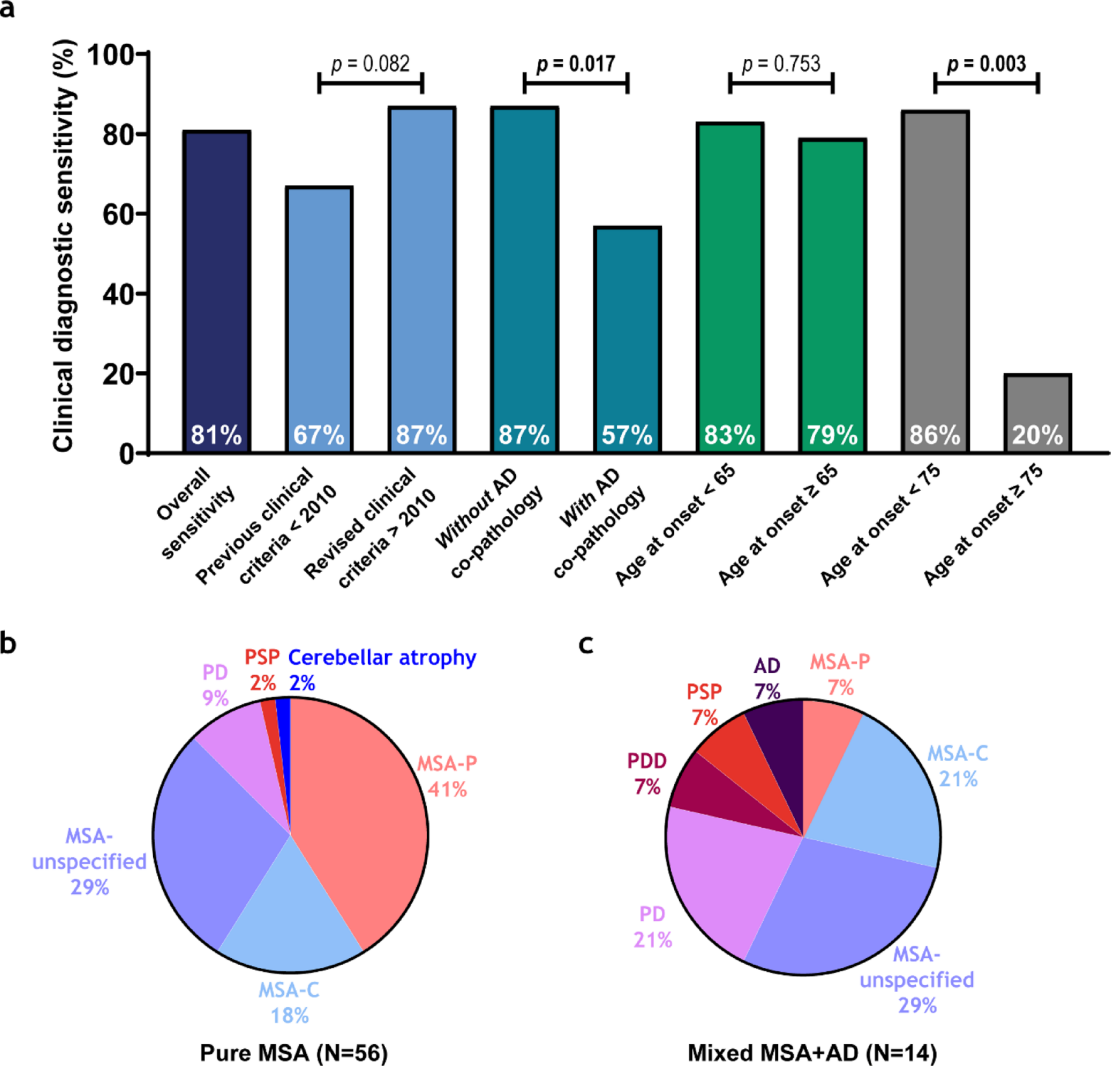
Diagnostic sensitivity is lower in mixed MSA + AD donors and late MSA disease onset. **a** Diagnostic sensitivity is visualized in a bar chart and represents the percentage of neuropathologically MSA donors that received a clinical antemortem MSA diagnosis. Diagnostic sensitivity is represented in each bar for overall sensitivity, before and after the revised MDS criteria [6], *without* and *with* AD co-pathology, for age at onset of MSA < 65 and ≥ 65 years of age, and for age at onset of MSA < 75 and ≥ 75 years of age, respectively. Groups were compared using a Fisher’s exact test for categorical variables. *p *values represent the differences between bars of the same color, *p *values in bold survived FDR correction. **b,c** Pie charts show final clinical antemortem diagnoses in pure MSA **(b)** and mixed MSA + AD **(c)**

The overall clinical diagnostic sensitivity in this neuropathologically confirmed MSA cohort indicates that 81% of the donors received an MSA diagnosis during life (Fig. 1). A clinical MSA-P diagnosis corresponded with striatum-predominant MSA pathology in 92%, while only 51% of all striatum-predominant MSA cases were classified as MSA-P during life. A clinical MSA-C diagnosis corresponded with cerebellum-predominant MSA pathology in 69% of the cases, but only 50% of cerebellum-predominant MSA donors were clinically recognized as MSA-C. When the clinical MSA subtype was not specified, donors had striatum-predominant (50%), cerebellum-predominant (35%), or mixed striatum = cerebellum (15%) pathology (Table 3).

**Table 3:** Correspondence between clinical MSA subtype and neuropathological confirmation

a. Clinical diagnosis	Total (*n*)	Pathological striatum- predominant MSA	Pathological cerebellum-predominant MSA	Pathological MSA-mix (striatum = cerebellum)
MSA-P	*24*	*22* (92%)	*0* (0%)	*2* (8%)
MSA-C	*13*	*0* (0%)	*9* (69%)	*4* (31%)
MSA-unspecified	*20*	*10* (50%)	*7* (35%)	*3* (15%)
Non-MSA	*13*	*11* (85%)	*2* (15%)	*0* (0%)

b. Pathological diagnosis	Total (*n*)	Clinical MSA-P	Clinical MSA-C	Clinical MSA-unspecified	Clinical Non-MSA
Striatum-predominant MSA	*43*	*22* (51%)	*0* (0%)	*10* (23%)	*11* (26%)
Cerebellum-predominant MSA	*18*	*0* (0%)	*9* (50%)	*7* (39%)	*2* (11%)
MSA-mix (striatum = cerebellum)	*9*	*2* (22%)	*4* (44%)	*3* (33%)	*0* (0%)

Neuropathological postmortem diagnoses were determined by the assessing neuropathologist according to criteria by Jellinger et al. [13]. Clinical diagnosis reflect the final antemortem diagnosis made by treating physician. **a** The percentage of clinical subtype diagnosis corresponding to postmortem diagnosis. **b** The percentage of neuropathologically confirmed diagnoses clinically recognized correspondingly

To assess potential changes in diagnostic practices over time, clinical diagnoses before and after 2010 were compared, allowing a two-year window for the adoption of revised clinical MSA criteria published in 2008 [6]. Sensitivity increased from 67 to 87% although this difference did not reach statistical significance (*p* = 0.082) (Fig. 1a). Diagnostic sensitivity was lower in donors with mixed MSA + AD pathology (57%), compared to those with pure MSA pathology (87%; *p* = 0.017). When we examined associations between diagnostic sensitivity and clinical outcomes, only age at death was significant (r = 0.35, *p* = 0.012); no associations were found with age at onset, disease duration, CDR score, or APOE-ε4. Among mixed MSA + AD donors, the clinical diagnoses included PD (21%), PDD, PSP, and AD (7% each). In pure MSA, the clinical diagnoses were PD (9%), PSP and cerebellar atrophy (2% each) (Fig. 1b).

A previous study reported reduced diagnostic sensitivity in late‐onset MSA using an age‐at‐onset threshold of 75 years [9]. In our cohort, application of this same cut‐off (*n* < 75 years = 65 vs. ≥ 75 years = 5) resulted in a decline in sensitivity from 86 to 20% (*p* = 0.003). To determine whether a more modest increase above the cohort mean onset age (60 years) would similarly affect diagnostic performance, we also evaluated a threshold of 65 years (*n* < 65 years = 46 vs. ≥ 65 years = 24), which revealed no significant change in sensitivity (*p* = 0.75) (see Fig. 1a). This data indicates that only very late‐onset disease (≥ 75 years) is associated with a substantial loss of diagnostic accuracy in MSA.

### Heterogeneity of proteinopathies

**Fig. 2:**
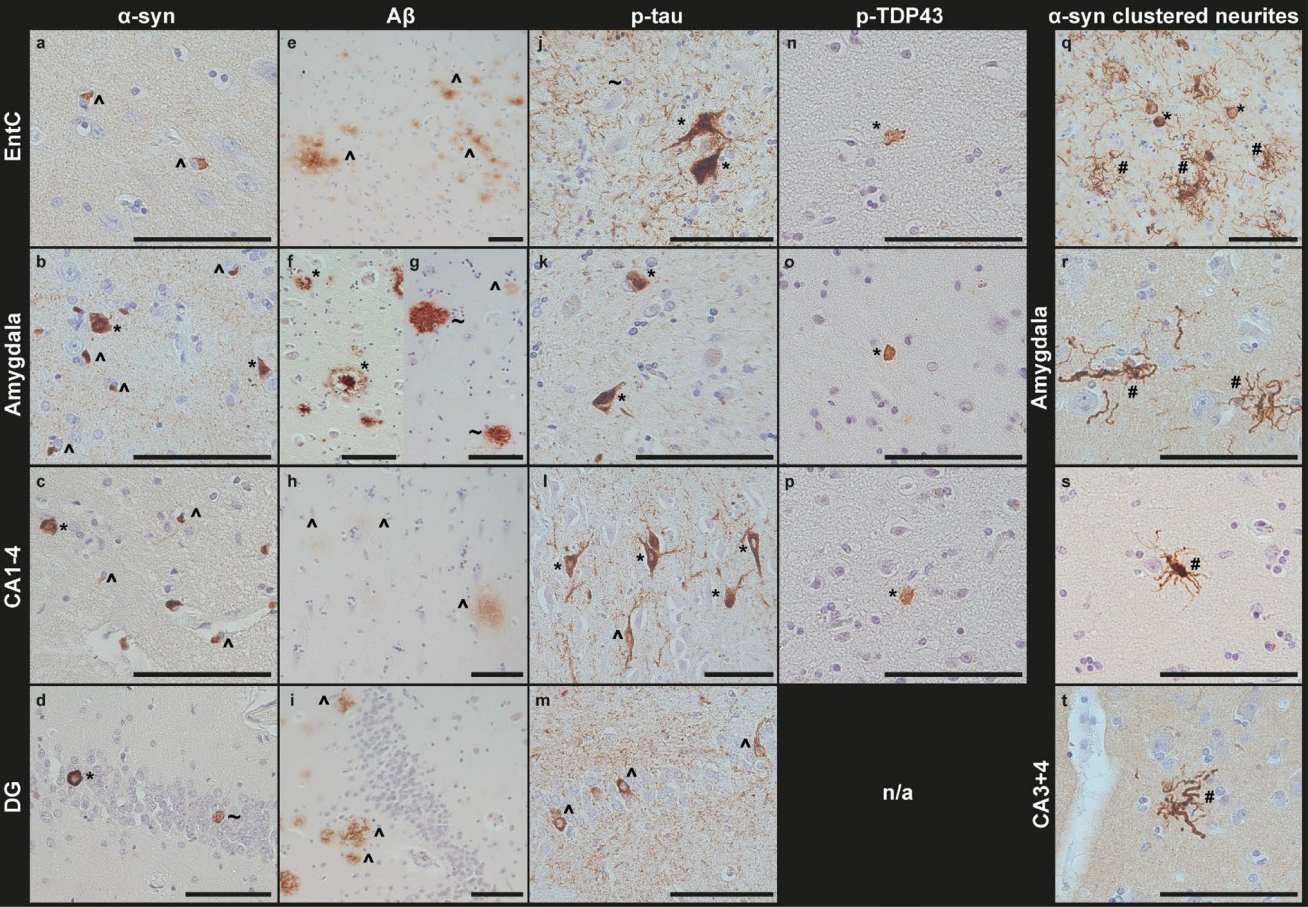
Regional patterns of α-syn, Aβ, and p-tau pathology in limbic regions in MSA. Representative images of α-syn (**a-d, q-t**), Aβ (**e-i**), p-tau (**j-m**), and pTDP-43 (**n-p**) across the EntC, amygdala, CA, and DG. **a** α-Syn pathology in the EntC is characterized by prominent GCIs (indicated by ^). **b** In the amygdala, α-syn pathology is heterogeneous, with a mixture of GCIs (^), neuronal inclusions (*) and α-syn neurites (not in this image). **c** α-Syn in CA regions, of which CA3 + 4 is depicted here, demonstrating both GCIs (^) and NCIs (*), while DG (**d**) contains mostly ring-shaped NCIs (*) and some diffuse NCIs ( ~) localized to the granule cell layer. **e** Aβ pathology is extensive in the EntC, with predominance of diffuse plaques (indicated by ^). **f,g** Besides diffuse plaques (^), the amygdala shows a high burden of compact ( ~) and classic-cored (*) plaques. In contrast, CA regions, of which CA1 is shown here, exhibit only sparse diffuse plaques (^) (**h**), and the DG is minimally affected, with a small number of diffuse (^) and some compact plaques (not in this image) surrounding the granule cell layer (**i**). p-Tau aggregates are mainly observed as threads (indicated by ~) and some NFTs (*) in the EntC (**j**) and amygdala (**k**), with relatively less NFTs compared to the other limbic regions. **l** CA1-4, of which CA3 + 4 is shown here, is characterized by widespread mature NFTs (*) and pretangles (^). **m** In DG mainly pretangles (^) were observed. **n-p** Examples of the sparse cytoplasmic pTDP-43 inclusions that were observed in the EntC (**n**), amygdala (**o**) and CA1 (**p**). **q-s** Examples of the distinct clusters of α-syn neurites that were observed in the amygdala of 8 donors (#2,4,9,14,21,51,61,68; donor #51 is shown in **q + r**, donor #2 in **s)** and in CA3 + 4 of 1 donor (donor #51). Scale bar in all images represents 100 µm

Morphologically, α-syn pathology displayed distinct regional profiles, with prominent GCIs in the EntC, a high diversity of morphological structures in the amygdala, including GCIs, neuronal inclusions and neurites, and a high density of both GCIs and neuronal inclusions in CA2 with moderate density in other CA regions (Fig. 2a-c). In the DG, α-syn pathology was more selective, with ring-shaped neuronal inclusions concentrated in the granule cell layer (Fig. 2d). Interestingly, 8 donors (#2, 4, 9, 14, 21, 51, 61, 68, see Online Resource 1, part 1) showed distinct α-syn pathology shaped like clustered neurites in the amygdala (Fig. 2q-s**)**. In one donor, this type of α-syn pathology in the amygdala was extensive and was also observed in CA3 + 4 (#51) (Fig. 2t). Among these 8 donors, no clear patterns or differences in phenotype regarding age, Braak α-syn, Braak NFT, Thal Aβ, SND, OPCA, TDP-LATE, CAA, CERAD, ARTAG stage, or APOE status were found when compared to donors without this type of α-syn clusters in the amygdala.

Aβ plaques were most pronounced in the EntC and amygdala, manifesting primarily as diffuse plaques in the EntC with an addition of compact and classic-cored plaques in the amygdala (Fig. 2e-g). The CA regions and DG showed limited Aβ pathology, consisting mainly of diffuse and occasionally compact plaques (Fig. 2h, i). p-Tau pathology appeared primarily as threads in both the EntC and the amygdala, with tangles present but less frequently than the thread pathology (Fig. 2j, k). The CA regions showed many tangles, mainly consisting of mature NFTs in CA2 together with pretangles in other CA regions (Fig. 2l). In the DG, mainly pretangles were observed (Fig. 2m). Sparse cytoplasmic pTDP-43 inclusions (< 2 per limbic region) were observed in the EntC, amygdala and CA1-4 (Fig. 2n-p). Differences between pure MSA and mixed MSA + AD donors are not depicted, as α-syn pathology was similar between groups, and Aβ and p-tau pathology, though largely restricted to mixed donors by definition, displayed comparable regional distribution patterns.

### Prevalence and regional distribution of pathologies

**Fig. 3:**
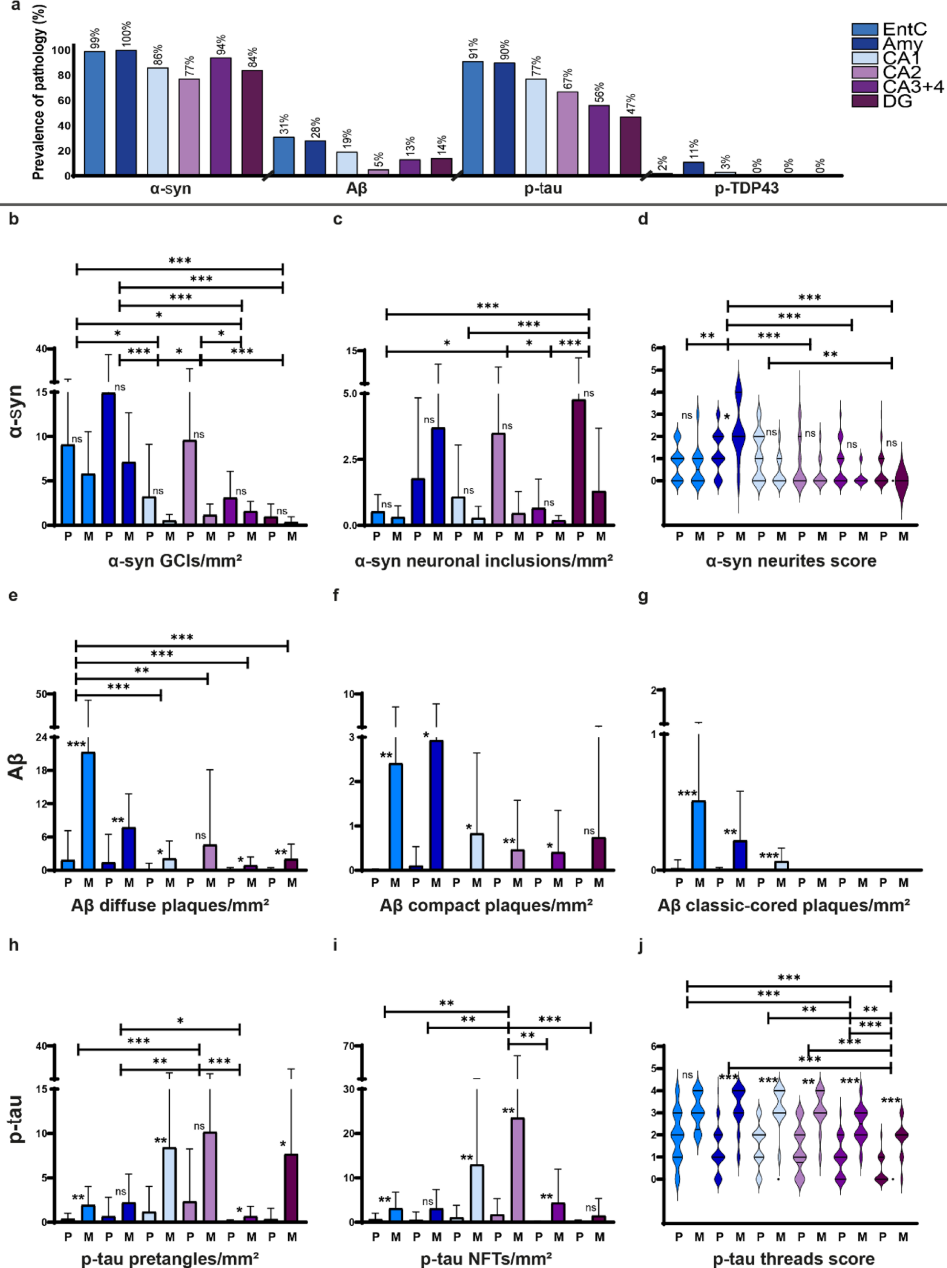
Prevalent neuronal α-syn in the amygdala and DG, diffuse but also compact and classic-cored Aβ plaques in the EntC and amygdala and mostly p-tau threads, NFTs and some pretangles in CA regions. (**a**) Boxplots show the prevalence (% of donors affected) of α-syn, Aβ, p-tau and pTDP-43 pathology in each brain region. Regional color codes in (**a**) are used consistently throughout the figure. (**b, c, e-i)** Bar graphs show mean inclusion densities per mm^2^ [SD]. (**f, n**) Violin plots represent ordinal scores for neuritic α-syn (**d**) and p-tau threads (**j**). α-Syn inclusion density was largely similar between groups **(b-d)**, except for a higher threads score in the amygdala of mixed MSA + AD donors (**d**). GCIs, and neurites were most prominent in the amygdala (**b, d**), and neuronal inclusions in the amygdala and DG (**c**). By design, mixed MSA + AD donors showed higher Aβ and p-tau pathology across several limbic regions (**e-j**). Diffuse Aβ plaques predominated, with highest Aβ plaque densities in the EntC and amygdala (**e–g**). p-tau predominantly consisted of mature NFTs, with highest tangle density in CA2 and CA1 (**h-j**). Significant differences between pure MSA (P) and mixed MSA + AD (M) are indicated between bars. Regional differences are marked with capped lines. Significance is demonstrated as * *p* < 0.05, ** *p* < 0.01, *** *p* < 0.001, ns = not significant

α-Syn pathology was detected in 100% of all donors in the amygdala, 99% in the EntC, 94% in CA3 + 4, 86% in CA1, 84% in the DG, and 77% in CA2 (Fig. 3a). Aβ pathology in 31% of the donors in the EntC, 28% in the amygdala, 19% in CA1, 14% in the DG, 13% in CA3 + 4, and 5% in CA2. p-Tau in 91% of the donors in the EntC, 90% in the amygdala, 77% in CA1, 67% in CA2, 56% in CA3 + 4, and 47% in the DG. Lastly, pTDP-43 pathology was detected in only 11% of the donors in the amygdala, 3% in CA1, 2% in the EntC, and was absent in CA2, CA3 + 4 and the DG. Due to the low number of pTDP-43 inclusions (maximum 2 per region), this pathology was not further quantified.

Inter-rater reliability of pathological burden between assessors was as following for α-syn (ICC2: 0.97, MAE: 0.44), Aβ (ICC2: 0.99, MAE 0.21) and p-tau (ICC2: 0.96, MAE: 0.31). The most prominent findings on the differences in regional distribution of α-syn, Aβ, and p-tau pathology between groups and regions were described below (Fig. 3b-j), and full pairwise comparisons are reported in Online Resource 1, part 8 (groups), and 9 (regions).

Mixed MSA donors showed higher α-syn threads scores in the amygdala (*p* = 0.012), while total α-syn %area, GCIs and neuronal α-syn did not differ between groups (Fig. 3b-d). GCIs and α-syn neurites were most abundant in the amygdala (vs. EntC, CA1, CA3 + 4 and DG, all *p* ≤ 0.019 and vs. EntC, CA2, CA3 + 4 and DG, all *p* ≤ 0.002; respectively), while neuronal α-syn predominated in the DG (vs. EntC, CA1 and CA3 + 4, all *p* < 0.001). As per definition, Aβ and p-tau burden was higher in mixed MSA + AD donors in multiple regions (Fig. 3e-j). The highest density of diffuse Aβ plaques was observed in the EntC (vs. CA1-4 and DG, *p* ≤ 0.003), compact Aβ plaques were similarly distributed across regions, and classic-cored Aβ plaques were limited to the EntC, amygdala and CA1. For p-tau, the highest densities of pretangles (vs. EntC, amygdala and CA3 + 4, *p* ≤ 0.005) and mature NFTs (vs. EntC, amygdala, CA3 + 4 and DG, *p* ≤ 0.004) were observed in CA2, while most threads were observed in the EntC (vs. CA3 + 4 and DG, both *p* < 0.001).

### Regional vulnerability underlies co−pathology patterns in MSA

**Fig. 4:**
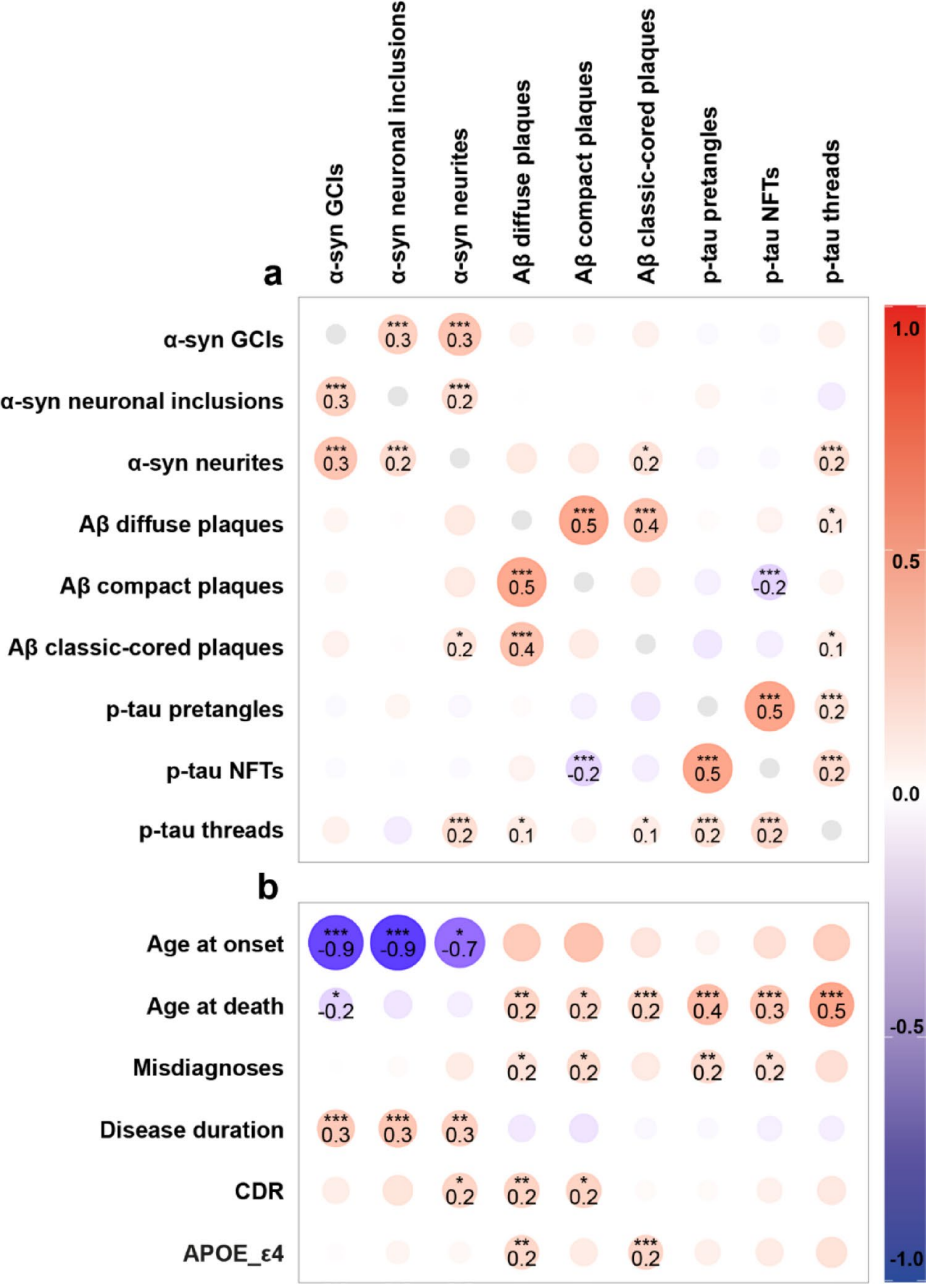
Neuropathological and clinicopathological associations across limbic brain regions. Bubble plots show standardized effect estimates from linear models assessing associations across regions (EntC, amygdala, CA1, CA2, CA3 + 4 and DG) between neuropathological burden and clinical variables, adjusting for age at death and sex. Pairwise associations between standardized neuropathological burden (**a**), and associations between clinical variables and neuropathological burden (**b**). α-Syn pathology was not associated with Aβ or p-tau pathology. In contrast, co-pathologies (Aβ and p-tau) were linked to older age at death, clinical misdiagnoses, and higher CDR scores. Notably, higher CDR scores were associated with neuritic, but not glial, α-syn pathology, suggesting a cognitive contribution of neuronal α-syn burden alongside co-pathology in MSA. Bubble size represents the absolute strength of the correlation, color indicates the direction (red = positive, white = no association, blue = negative). Significance after FDR correction is demonstrated in bold as *p < 0.05, **p < 0.01, and ***p < 0.001

To explore potential shared mechanisms between proteinopathies in MSA, we examined standardized regression coefficients between neuropathological inclusions, adjusted for age at death and sex (Fig. 4; Online Resource 1, part 10a + b). Since %area of α-syn, Aβ and p-tau showed minimal differences in regional distribution, we examined associations for morphological outcome measures only.

Overall, α-syn pathology, including GCIs, neuronal inclusions and neurites, did not show a strong correlation with co-pathology. Notable exceptions included associations between α-syn threads and both classic-cored Aβ plaques (r ≥ 0.16, *p* = 0.015) and p-tau threads (r = 0.18, *p* < 0.001). Additionally, GCI density associated with neuronal α-syn inclusions and neurites (r ≥ 0.26, both *p* < 0.001). A negative association was observed between compact Aβ plaques and p-tau NFTs (r = -0.20, *p* < 0.001), while diffuse Aβ plaques were positively associated with p-tau threads (r = 0.11, *p* = 0.015), and higher Aβ Thal phases associated with p-tau NFTs and threads (r = 0.23, *p* = 0.009 and r = 0.24, *p* = 0.029). Lastly, TDP-LATE stage was associated with classic-cored Aβ plaques, p-tau pretangles and NFTs (r ≤ 0.14, *p* ≤ 0.036) and not with α-syn pathology. See Online Resource 1, part 10a + b for all neuropathological associations.

To examine whether these associations reflect consistent patterns or regional vulnerability, we next conducted region-specific analyses across the six limbic regions (EntC, amygdala, CA1, CA2, CA3 + 4 and DG) [Online Resource 1, part 11]. α-Syn pathology did not associate with Aβ or p-tau pathology in any individual region. However, GCIs remained significantly associated with neuronal α-syn inclusions and neurites, with the strongest associations in CA1 (r = 0.92 and r = 0.54, both *p* < 0.001). Pronounced regional vulnerability was observed in CA3 + 4, which exhibited the strongest correlations between co-pathologies; diffuse and compact Aβ plaques associated with p-tau pretangles, NFTs and threads in CA3 + 4 (all r ≥ 0.39, *p* ≤ 0.002).

### Cognitive impairment in MSA reflects neuronal α−syn, Aβ, and p−tau pathology burden

To assess how limbic pathological burden influences clinical presentation, we examined associations between pathological inclusions and clinical outcomes across all regions (Fig. 4; Online Resource 1, part 10c + d). α-Syn GCIs, neuronal inclusions, and neurites were associated with a younger age at onset (r ≥ -0.70, *p* ≤ 0.021) and a longer disease duration (r ≥ 0.26, *p* ≤ 0.007), while Aβ and p-tau pathology were not. A higher age at death was associated with diffuse, compact and classic-cored Aβ plaques (all r ≥ 0.23, *p* ≤ 0.020), as well as p-tau pretangles, NFTs and threads (all r ≥ 0.33, *p* < 0.001). Higher CDR scores were linked to α-syn neurites (r = 0.24, *p* = 0.027) and to diffuse and compact Aβ plaques (both r ≥ 0.24, *p* ≤ 0.015). Furthermore, a higher rate of misdiagnoses was observed in donors with compact Aβ plaques (r = 0.21, *p* = 0.040) and p-tau pretangles and NFTs (both r ≥ 0.18, *p* ≤ 0.020). The associations with misdiagnoses remained significant after adjusting for age at onset (all r ≥ 0.20 for all, *p* ≤ 0.026). Diffuse and classic-cored Aβ plaques were associated with a higher frequency of APOE-ε4 alleles (r ≥ 0.22, *p* ≤ 0.002), but not with any of the other pathological inclusions. Although hallucinations appeared somewhat more frequently in pure MSA than in MSA + AD, we found no association between hallucinations and neuronal α-syn inclusions in limbic regions (r = –0.00, *p* = 0.970). The presence of hallucinations was also not associated with the severity of other pathological markers (r <  ± 0.13, *p* > 0.45) and was therefore not visualized in the graph. Lastly, Braak α-syn, MSA-SND, and MSA-OPCA stages were not associated with any of the clinical outcome measures. See Online Resource 1, part 10d for all associations between neuropathological stages and clinical outcomes.

To determine whether the clinicopathological associations reflected general or region-specific clinical vulnerability, we explored associations across limbic regions [see Online Resource 1, part 11]. Pathologies that associated most strongly with both age at onset and disease duration were α-syn GCIs and neuronal inclusions in CA1 (age at onset: both r ≥ -0.51, *p* < 0.001; disease duration: both r ≥ 0.55, *p* < 0.001). Pathology correlating most strongly with age at death included p-tau pretangles, NFTs and threads across all regions (all r ≥ 0.34, *p* ≤ 0.020) and diffuse Aβ plaques in the EntC, amygdala, CA1 and DG (r ≥ 0.32, *p* ≤ 0.025). Most strongly associated with CDR scores were diffuse and compact Aβ plaques in the EntC (both r ≥ 0.50, *p* < 0.001), p-tau pretangles in CA3 + 4 (r = 0.43, *p* = 0.002), and α-syn neuronal inclusions and neurites in the amygdala (r ≥ 0.54, *p* < 0.001). Pathologies strongly associated with misdiagnoses were p-tau pretangles in CA3 + 4 (r = 0.42, *p* = 0.002), p-tau NFTs (r = 0.41, *p* = 0.003) and neuronal α-syn inclusions in the amygdala (r = 0.32, *p* = 0.025), compact Aβ plaques in CA1 (r = 0.32, *p* = 0.025) and diffuse Aβ plaques in the DG (r = 0.36, 0.011). Lastly, diffuse and classic-cored Aβ plaques, together with p-tau pretangles and NFTs associated with APOE-ε4 allele frequency in the EntC (all r ≥ 0.31, *p* ≤ 0.034).

## Discussion

This study provides a comprehensive clinicopathological assessment of α-syn, Aβ, p-tau and pTDP-43 pathologies in a well-characterized cohort of 70 neuropathologically confirmed MSA donors, in which we emphasized their limbic regional patterns, morphological characteristics, and clinical relevance. While α-syn GCI pathology remains the defining hallmark of MSA [12], our findings emphasize that limbic co-pathologies and neuronal α-syn inclusions are both common and clinically meaningful, particularly in relation to cognitive symptoms and diagnostic accuracy. Unlike previous studies that mainly reported the pathological prevalence or Braak and Thal staging originally developed for PD or AD, we provide a systematic quantitative and morphological assessment of co-pathologies in limbic regions of MSA and link these directly to clinical outcomes.

Although only 20% of the included donors met the criteria for mixed MSA + AD pathology (Thal Aβ or Braak NFT stage ≥ 3), a substantial proportion of pure MSA cases exhibited some degree of Aβ or p-tau pathology as reflected by 31% of the total cohort showing Aβ and 91% showing p-tau inclusions in the EntC. This is consistent with prior studies reporting low-to-intermediate AD co-pathology in MSA, particularly among older individuals [22–24, 28]. The age-related, high prevalence of p-tau inclusions suggests that tauopathy is not uncommon in MSA and may arise as part of a general aging process. Likewise, Aβ plaque formation was associated with age at death in our cohort. A large, unselected autopsy series spanning ages 1–100 years (*n* = 2332) provided a population‐based estimate of age‐related AD changes independent of neurological diagnosis [47]. Among donors aged 51–70 years (the mean age was 60 in our cohort), 18% exhibited Braak NFT stage ≥ 3 and 5% showed Thal Aβ ≥ 3. These prevalences closely mirror the observations in our MSA cohort (14 and 16%, respectively), reinforcing the interpretation that most AD co-pathology in MSA reflects background aging. Two cases in our cohort demonstrated a discrepancy between AD markers (high Thal Aβ but low Braak NFT stages), consistent with an Aβ-predominant phenotype previously described within MSA [48]; both individuals carried the APOE-ε4 allele.

Mixed MSA + AD donors were significantly older at disease onset and death, carried the APOE-ε4 allele more frequently, and had a higher CAA and CERAD burden than pure MSA donors. These group differences support the hypothesis that advanced age and genetic risk factors (notably APOE- ε4) could contribute to the accumulation of co-pathologies in neurodegeneration in general [20, 24]. In MSA, higher Thal Aβ phases were also described in donors carrying the APOE-ε4 allele [28]. From a clinical perspective, assessment of APOE-ε4 genotype and age at onset may stratify patients by the likelihood of AD co-pathology developing in MSA and therefore be of importance for future clinical trials.

In our cohort, the clinical sensitivity for detecting MSA, which is the proportion of pathologically confirmed cases correctly diagnosed during life, was 81%, consistent with prior studies [7, 9–11]. The predominant pathology was striatonigral degeneration in 61% of donors, aligning with reports of a higher prevalence of MSA-P in Western populations [9, 12]. When a clinical subtype was assigned during life, concordance with the predominant pathology was strong: 92% of clinical MSA-P cases matched striatum-predominant pathology, and 69% of MSA-C cases matched cerebellum-predominance. Despite these high concordance rates, only approximately half of neuropathologically confirmed donors were clinically assigned a subtype concordant with the predominant pathology, reflecting the limitations of in vivo classification. Notably, the clinical distinction between MSA-P and MSA-C is not an end in itself as substantial symptom overlap often complicates subtype differentiation in practice. Importantly, positive predictive value (PPV), the probability that a clinical diagnosis reflects MSA pathology, differs conceptually from sensitivity. One study in the US reported PPVs of 91% for MSA-C and 64% for MSA-P [49], but to our knowledge, no study has previously assessed the accuracy of clinical subtype assignment.

Clinicopathological overlap between striatonigral and olivopontocerebellar features was extensive: only three donors had isolated striatonigral pathology without any olivopontocerebellar involvement, while all others showed some degree of mixed pathology. Correspondingly, clinical symptoms also showed substantial overlap: parkinsonism and cerebellar symptoms were reported in 90% and 85% of the cohort, respectively. To further support these findings, we did not observe differences in the distribution of co-pathologies between striatum and cerebellum, predominant cases, as previously described [28]. These results suggest that the strict binary classification into MSA-P and MSA-C may oversimplify the underlying disease complexity [30].

Interestingly, the three isolated striatonigral cases all had short disease durations (3–4 years), and two died from respiratory complications. This raises the possibility that mixed striatal + cerebellar pathology evolves over time, and that early mortality in these cases may have prohibited broader pathological involvement. Indeed, an earlier autopsy series of 100 MSA patients even consisted exclusively of mixed (striatal + cerebellar pathology) donors without any pure cases, supporting this temporal evolution [30]. Moreover, histological analyses have shown that α-syn deposition in striatonigral and olivopontocerebellar regions progressively converges as disease duration increases, further supporting a temporal evolution from isolated to mixed pathology [50]. In addition, the observed association between α-syn inclusions (both GCIs and NCIs) and younger age at onset may reflect a more aggressive disease phenotype. Notably, longer disease duration was linked to higher α-syn burden, potentially reflecting progressive α-syn accumulation over time. An early study suggested that GCIs may exert more influence on olivopontocerebellar pathology, whereas striatonigral degeneration may involve a rapid, GCI-independent process of neuronal loss [30]. In our cohort, we could not confirm this since we did not find an association between GCIs or neuronal inclusions and MSA-SND or OPCA stages. However, these data imply that age at onset and disease duration modulate the pathological spectrum, underscoring the value of considering early- versus late-onset MSA in both clinicopathological classification and trial design.

After the implementation of the revised criteria for clinical diagnosis of MSA, introduced in 2008 [6], diagnostic sensitivity improved from 67 to 87% in this autopsy cohort. However, the presence of mixed pathology and cognitive symptoms continued to obscure clinical diagnosis, particularly in older individuals. Notably, misdiagnosis rates in our cohort rose to 80% when symptom onset occurred after age 75, indicating that while moderate aging alone does not markedly impair diagnostic sensitivity, very late disease onset severely confounds clinical recognition. Crucially, the 2008 consensus criteria listed onset beyond 75 years as a non-supportive feature [6], a restriction removed in the 2022 criteria in recognition of the fact that MSA can manifest at advanced ages [1]. Only three donors in this cohort were evaluated after the publication of the 2022 criteria [1], prohibiting meaningful analysis of their impact. The 2022 revision also incorporated characteristic MRI signs, objective autonomic measurements, and allowed dementia unless it occurred within 3 years of onset, whereas the 2008 criteria used dementia as absolute exclusion criterion [51]. Although initial validation of the 2022 criteria suggested improves specificity without loss of sensitivity [51], a recent autopsy study of 240 cases found that including MRI features reduced sensitivity without improving specificity [52], suggesting limited diagnostic value of MRI. Taken together, these data highlight how both aging and diagnostic criteria limitations continue to challenge diagnostic accuracy in MSA.

Beyond these age-related factors, we observed that a higher p-tau and Aβ plaque burden was associated with increased misdiagnosis rates, even after adjusting for age at onset. These donors were more often misclassified as having PD, PDD, PSP, or AD. Nevertheless, the core features of MSA—parkinsonism, cerebellar dysfunction, and autonomic failure—did not differ between pure and mixed cases, suggesting that AD co-pathology may modulate clinical phenotype, but does not change MSA core symptoms. Region-specific analyses linked misdiagnoses to p-tau pathology in the amygdala and CA3 + 4, Aβ plaques in CA1 and the DG, and neuronal α-syn inclusions in the amygdala, identifying potential anatomical substrates of diagnostic error. Similarly, in dementia with Lewy bodies (DLB), higher Braak NFT stages have been shown to reduce diagnostic accuracy [53]. One study in MSA reported lower diagnostic sensitivity in patients with comorbid pathologies though this was partly attributed to age at onset [28]. Our data extend these findings by showing that limbic co-pathologies, independent of age, significantly contribute to diagnostic error, alongside the additive effect of very late onset itself.

In our cohort, cognitive impairment, as reflected by elevated CDR scores, was significantly associated with Aβ plaques in the EntC, p-tau pathology in CA3 + 4 and neuronal α-syn inclusions in the amygdala. Prior MSA autopsy studies that focused on Aβ Thal and NFT Braak stages instead of regional vulnerability reported conflicting results. A small series (*n* = 18) and a large MSA cohort (*n* = 160) found no difference in Aβ Thal or NFT Braak stages between cognitively impaired and unimpaired donors [28, 54], whereas another series (*n* = 44) reported that only the cognitively impaired cases (4/44) had Aβ Thal and NFT Braak stages of 3 or higher while none of the unimpaired cases did [55]. These discrepancies likely reflect the variable definitions of cognitive impairment used in these cohorts, from MMSE scores < 28 [54], to physician or family member impression [28], to retrospective chart review [55], underscoring the need for standardized neuropsychological evaluation in clinical MSA cohorts. Evidence linking regional neuronal α-syn pathology to cognition remains limited. One study reported more neuronal α-syn inclusions in the DG of cognitively impaired cases, but no difference in the amygdala [31], while a case report described extensive neuronal α-syn pathology in the DG and amygdala in an MSA donor with dementia [56]. Our findings suggest that cognitive impairment in MSA reflects contributions from both limbic Aβ and p-tau co-pathologies and neuronal α-syn in the amygdala, highlighting the necessity to consider both lesion types in research perspectives. Moreover, Aβ and p-tau biomarkers are already used in clinical practice and may offer additional insights into the in vivo relevance of co-pathology in MSA [57]. Taken together, these observations underscore the imperative to reconceptualize cognitive impairment in MSA not as an exclusionary ‘red flag’ but rather as a contributory element of disease burden, and to refine diagnostic frameworks that embrace the full clinical and pathological heterogeneity of MSA.

Morphological analyses revealed distinct region-specific vulnerability across limbic structures. The amygdala was enriched for all major morphological types of α-syn, Aβ, and p-tau pathology, whereas CA2 showed a high density of p-tau tangles and both glial and neuronal α-syn inclusions. Although the amygdala is recognized as a key hub for proteinopathies in neurodegenerative diseases [33], detailed studies of its pathology in MSA are lacking, precluding direct comparison with our findings. Recent evidence has demonstrated notable astrocytic α-synuclein accumulation in the amygdala in MSA [58], underscoring the need for a broad antibody panel to distinguish astrocytic from oligodendroglial inclusions in our cohort. A study evaluated pathological accumulation patterns in the hippocampus and likewise reported that p-tau tangle density in CA2 in both MSA and AD, and α-syn inclusions (type unspecified) in CA2 were elevated in MSA [59]. The DG, by contrast, displayed selective α-syn NCIs with a ring-shaped morphology, as previously described in MSA [60]. Importantly, it should be noted that the use of a C-terminal KM51 antibody for α-syn may underestimate the burden of neuronal α-syn pathology, which was recently described to be better detected by N-terminal antibodies in synucleinopathies [61]. In addition, we found a distinct type of α-syn pathology in 8 (11%) MSA donors, which was mostly limited to the amygdala and presented as focal clusters of neurite pathology. This type of α-syn pathology could not be linked with any distinctive clinical or pathological features. To our knowledge, this specific amygdala-localized α-syn pathology has not been previously described in MSA; thus, future studies using double immunostaining for astrocytic, neuronal, and oligodendroglial markers will be essential to define the cellular origin and pathological significance of these inclusions.

The association between Aβ and p-tau pathology in MSA did not follow a unidirectional pattern. While diffuse Aβ plaques and higher Thal stages correlated positively with tau NFTs and threads, compact plaques showed a negative association with p-tau NFTs in our cohort. Previous studies have described the sequential maturation of p-tau pathology from pretangles to mature NFTs [62], and the progression of Aβ deposition from diffuse to more compact or classic-cored plaques [47]. In AD, diffuse plaques typically appear early, followed by p-tau accumulation and the emergence of compact plaques in later stages [63]. This bidirectional pattern in MSA may reflect a temporal dissociation between early tauopathy and late-stage Aβ plaque maturation, or alternatively, a decline in detectable tau pathology in the context of advanced neurodegeneration [64].

Despite frequent co-occurrence of α-syn, Aβ, and p-tau pathologies in limbic regions, our findings do not support robust synergistic interactions between these protein aggregates in MSA. α-Syn burden did not correlate with Aβ or p-tau pathology across any limbic region, suggesting limited neuropathological convergence. Instead, positive correlations between α-syn GCIs and NCIs, most notably in the CA1, are more likely attributable to the anatomical co-occurrence of these inclusions and resulting regional susceptibility. A modest correlation between α-syn threads and tau threads likely reflects spatial overlap rather than a mechanistic interaction.

The principal strength of this study lies in its novel, systematic evaluation of diverse pathological inclusions and their morphologies within limbic regions in a well-characterized MSA cohort. By correlating morphological pathology with clinical data, we provide crucial insights into clinicopathological relationships and unveil patterns of disease phenotypes that have remained previously unappreciated. This autopsy study also has its limitations. Owing to its retrospective design, some clinical measures may have been underscored, and euthanasia in 30 out of 70 donors may have affected estimates of disease duration and end-stage clinical features. Methodologically, the use of a C-terminal KM51 antibody for α-syn may have led to an underestimation of the burden of neuronal α-syn pathology [61], and the use of AT8 and 6F/3D antibodies for p-tau and Aβ, respectively, could have failed to detect certain species of these proteins [65, 66]. Future studies should incorporate an expanded panel of α-syn, p-tau and Aβ antibodies to improve the accuracy of cellular pathology assessment, alongside a comprehensive set of systematically collected clinical outcome measures aligned with diagnostic criteria.

## Conclusion

This study demonstrates that limbic co-pathologies, particularly Aβ and p-tau, and neuronal α-syn are prevalent and clinically relevant in a substantial proportion of MSA cases. These co-pathologies not only correlate with cognitive decline but also contribute to a reduction in diagnostic accuracy, especially in very late-onset cases, and are further modulated by APOE-ε4 status. Our data support the removal of age-related and cognitive exclusion criteria in the 2022 MDS framework, yet simultaneously acknowledge the ongoing difficulty of accurately recognizing mixed pathology in vivo. Incorporation of co-pathological burden into diagnostic frameworks and clinical trial designs using in vivo measures of AD co-pathology is therefore crucial. Future prospective investigations that integrate precise neuropathological quantification, systematic neuropsychological evaluation, APOE stratification, and advanced in vivo measures of co-pathology will be essential to elucidate the interplay between α-syn, p-tau, and Aβ in MSA and to inform the development of interventions targeting both the primary synucleinopathy and its limbic co-pathological modifiers. Ultimately, these efforts may pave the way for targeted therapies that address the full pathological spectrum in MSA.

## Supplementary Information

Below is the link to the electronic supplementary material.

Supplementary file1 (DOCX 21046 KB)

## Data Availability

Supporting data include supplementary tables, supplementary figures, and supplementary material. Accessibility to raw outcome measures will be made available on reasonable request.
