# Supplementary material for: Limbic Alzheimer’s co-pathology in multiple system atrophy is associated with cognitive impairment and diagnostic inaccuracy
Source: Acta Neuropathol. 2025 Sep 18;150(1):30. doi: 10.1007/s00401-025-02940-0 (PMC12446110; doi:10.1007/s00401-025-02940-0)
Supplement: Supplementary file 1 — Supplementary file1 (DOCX 21046 KB) [file 401_2025_2940_MOESM1_ESM.docx]

| **1. Case summaries of demographic and pathological features of MSA cohort** | | | | | | | | | | | | | | | | | | |
| --- | --- | --- | --- | --- | --- | --- | --- | --- | --- | --- | --- | --- | --- | --- | --- | --- | --- | --- |
| **Donor** | **Neuropathological predominant MSA subtype** | **Cohort group** | **Sex** | **Age at death (y)** | **Post-mortem delay (hours:mins)** | **Braak α-syn stage [1]** | **Braak NFT stage [2]** | **Thal Aβ phase [3]** | **MSA-SND stage [4]** | **MSA-OPCA stage [4]** | **TDP LATE stage [5]** | **CAA type [6]** | **CAA stage** | **CERAD [7]** | **ARTAG [8]** | **Argyrophilic grain disease (AGD) [9]** | **Brain weight (g)** | **APOE-ε genotype** |
| **1** | Striatum-predominant | mixed MSA+AD | F | 84 | 6:10 | 0 | 1 | 3 | 3 | 0 | N/a | 2 | 2 | N/a | No | Yes | 1175 | 2/2 |
| **2** | Striatum-predominant | pure MSA | F | 63 | 10:55 | 0 | 1 | 1 | 3 | 1 | 0 | 0 | 0 | none | No | Not mentioned | 1262 | 3/3 |
| **3** | Striatum-predominant | pure MSA | M | 57 | 9:45 | 0 | 1 | 1 | 3 | 2 | 0 | 0 | 0 | sparse | No | Not mentioned | 1297 | 3/4 |
| **4** | Striatum-predominant | pure MSA | M | 55 | 8:40 | 0 | 1 | 0 | 2 | 0 | 0 | 0 | 0 | none | No | Not mentioned | 1380 | 3/3 |
| **5** | Striatum-predominant | pure MSA | F | 84 | 4:45 | 0 | 2 | 0 | 3 | 0 | 0 | 0 | 0 | none | No | Not mentioned | 1288 | 3/4 |
| **6** | Striatum-predominant | pure MSA | F | 72 | 6:16 | 0 | 1 | 2 | 2 | 1 | N/a | 0 | 0 | none | No | Not mentioned | 1102 | 2/3 |
| **7** | Striatum-predominant | pure MSA | F | 71 | 7:40 | 6 | 1 | 0 | 3 | 1 | 0 | 0 | 0 | none | No | Not mentioned | 1358 | 3/3 |
| **8** | Striatum-predominant | pure MSA | F | 66 | 8:05 | 0 | 0 | 0 | 3 | 1 | 0 | 0 | 0 | none | No | Not mentioned | 1005 | 3/3 |
| **9** | Striatum-predominant | pure MSA | F | 61 | 6:50 | 0 | 1 | 0 | 3 | 1 | 0 | 0 | 0 | none | No | Not mentioned | 1072 | 3/3 |
| **10** | Striatum-predominant | pure MSA | M | 66 | 4:55 | 0 | 1 | 1 | 3 | 1 | 0 | 0 | 0 | none | No | Not mentioned | 1416 | 3/3 |
| **11** | Striatum-predominant | mixed MSA+AD | M | 79 | 2:35 | 5 | 4 | 2 | 3 | 1 | 1a | 2 | 2 | frequent | No | Not mentioned | 1295 | 4/4 |
| **12** | Striatum-predominant | pure MSA | F | 57 | 5:30 | 5 | 1 | 0 | 3 | 1 | 0 | 0 | 0 | none | No | Not mentioned | 1375 | 3/3 |
| **13** | Striatum-predominant | mixed MSA+AD | F | 67 | 7:15 | 0 | 1 | 4 | 3 | 2 | 0 | 0 | 0 | sparse | Occasional | Not mentioned | 1244 | 3/4 |
| **14** | Striatum-predominant | mixed MSA+AD | M | 70 | 2:04 | 5 | 3 | 2 | 2 | 1 | 0 | 0 | 0 | none | No | Not mentioned | 1265 | 3/4 |
| **15** | Striatum-predominant | pure MSA | F | 70 | 8:10 | 0 | 2 | 2 | 3 | 2 | 0 | 1 | 3 | sparse | No | Not mentioned | 1460 | 4/4 |
| **16** | Striatum-predominant | pure MSA | F | 61 | 6:50 | 4 | 0 | 0 | 3 | 2 | 0 | 0 | 0 | none | No | Not mentioned | 1060 | 3/3 |
| **17** | Striatum-predominant | pure MSA | F | 61 | 5:55 | 5 | 1 | 0 | 3 | 1 | 0 | 2 | 1 | none | No | Not mentioned | 1409 | 3/4 |
| **18** | Striatum-predominant | mixed MSA+AD | M | 77 | 5:25 | 3 | 3 | 0 | 3 | 2 | 0 | 0 | 0 | none | Occasional | Not mentioned | 1290 | 2/3 |
| **19** | Striatum-predominant | mixed MSA+AD | F | 60 | 7:10 | 5 | 1 | 3 | 3 | 1 | 0 | 2 | 1 | sparse | Occasional | Not mentioned | 1285 | 3/4 |
| **20** | Striatum-predominant | pure MSA | M | 59 | 7:30 | 3 | 1 | 1 | 3 | 1 | 0 | 0 | 0 | none | No | Not mentioned | 1285 | 2/4 |
| **21** | Striatum-predominant | mixed MSA+AD | F | 83 | 4:10 | 3 | 1 | 4 | 3 | 1 | N/a | 2 | 1 | sparse | Severe | Not mentioned | 1175 | 4/4 |
| **22** | Striatum-predominant | mixed MSA+AD | F | 74 | 5:05 | 4 | 4 | 3 | 3 | 2 | 0 | 1 | 1 | sparse | Occasional | Not mentioned | 1240 | 3/4 |
| **23** | Striatum-predominant | pure MSA | F | 65 | 7:05 | 4 | 1 | 1 | 3 | 1 | 0 | 2 | 1 | none | No | Not mentioned | 1185 | 3/3 |
| **24** | Striatum-predominant | pure MSA | F | 49 | 4:15 | 6 | 0 | 0 | 3 | 2 | 0 | 0 | 0 | none | No | Not mentioned | 1120 | 3/3 |
| **25** | Striatum-predominant | pure MSA | F | 62 | 5:55 | 6 | 1 | 1 | 3 | 2 | 0 | 0 | 0 | none | No | Not mentioned | 1207 | 3/4 |
| **26** | Striatum-predominant | pure MSA | F | 59 | 5:55 | 3 | 2 | 1 | 3 | 1 | 0 | 0 | 0 | none | No | Not mentioned | 1375 | 3/3 |
| **27** | Striatum-predominant | pure MSA | F | 62 | 5:45 | 5 | 1 | 1 | 3 | 1 | 0 | 0 | 0 | none | Occasional | Not mentioned | 1100 | 3/4 |
| **28** | Striatum-predominant | pure MSA | F | 74 | 6:35 | 6 | 1 | 0 | 2 | 1 | 0 | 0 | 0 | none | No | Not mentioned | 1406 | 3/3 |
| **29** | Striatum-predominant | pure MSA | F | 72 | 8:25 | 3 | 2 | 2 | 2 | 1 | 0 | 0 | 0 | none | Occasional | Yes | 1400 | 3/3 |
| **30** | Striatum-predominant | pure MSA | M | 70 | 3:52 | 4 | 2 | 0 | 3 | 1 | 0 | 0 | 0 | none | No | Yes | 1135 | 3/3 |
| **31** | Striatum-predominant | mixed MSA+AD | M | 84 | 5:40 | 4 | 4 | 4 | 3 | 2 | 2 | 1 | 2 | sparse | Severe | Not mentioned | 1355 | 3/4 |
| **32** | Striatum-predominant | pure MSA | M | 61 | 7:00 | 5 | 1 | 0 | 3 | 1 | 0 | 0 | 0 | none | Occasional | Not mentioned | 1485 | 3/3 |
| **33** | Striatum-predominant | pure MSA | F | 60 | 4:35 | 4 | 2 | 1 | 3 | 1 | 0 | 0 | 0 | none | Severe | Not mentioned | 1170 | 3/4 |
| **34** | Striatum-predominant | pure MSA | F | 60 | 6:20 | 6 | 1 | 1 | 3 | 2 | 1a | 0 | 0 | none | No | Not mentioned | 1215 | 3/3 |
| **35** | Striatum-predominant | pure MSA | M | 72 | 8:00 | 5 | 1 | 1 | 2 | 1 | 0 | 1 | 1 | none | Occasional | Not mentioned | 1400 | 2/4 |
| **36** | Striatum-predominant | pure MSA | F | 69 | 5:20 | 4 | 0 | 0 | 3 | 1 | 0 | 0 | 0 | none | No | Not mentioned | 1095 | 3/3 |
| **37** | Striatum-predominant | pure MSA | F | 74 | 9:20 | 5 | 2 | 2 | 3 | 2 | 1a | 2 | 1 | sparse | Occasional | Not mentioned | 1267 | 3/3 |
| **38** | Striatum-predominant | pure MSA | M | 68 | 4:45 | 6 | 1 | 2 | 3 | 2 | 0 | 1 | 2 | none | No | Not mentioned | 1590 | 3/4 |
| **39** | Striatum-predominant | pure MSA | F | 65 | 10:55 | 6 | 1 | 0 | 3 | 1 | 0 | 2 | 1 | none | No | Not mentioned | 1480 | 3/4 |
| **40** | Striatum-predominant | mixed MSA+AD | F | 67 | 5:20 | 5 | 3 | 4 | 3 | 1 | 1a | 2 | 1 | sparse | No | Not mentioned | n.a. | 4/4 |
| **41** | Striatum-predominant | pure MSA | F | 52 | 6:30 | 5 | 1 | 1 | 3 | 2 | 0 | 2 | 1 | none | No | Not mentioned | n.a. | 3/3 |
| **42** | Striatum-predominant | pure MSA | M | 54 | 5:40 | 5 | 0 | 0 | 3 | 1 | 0 | 0 | 0 | none | Occasional | Not mentioned | 1450 | 3/3 |
| **43** | Striatum-predominant | pure MSA | M | 60 | 6:45 | 6 | 1 | 0 | 3 | 2 | 0 | 0 | 0 | none | No | Not mentioned | n.a. | 2/4 |
| **44** | Cerebellum-predominant | pure MSA | F | 57 | 13:15 | 0 | 0 | N/a | 1 | 3 | N/a | 0 | 0 | none | No | Not mentioned | 1298 | 3/4 |
| **45** | Cerebellum-predominant | pure MSA | F | 59 | 20:35 | 0 | 1 | 0 | 2 | 3 | 0 | 0 | 0 | N/a | No | Not mentioned | 1199 | 3/4 |
| **46** | Cerebellum-predominant | pure MSA | M | 66 | 6:30 | 3 | 1 | 0 | 1 | 2 | 0 | 0 | 0 | none | No | Not mentioned | 1363 | 2/3 |
| **47** | Cerebellum-predominant | pure MSA | M | 67 | 6:10 | 3 | 2 | 0 | 1 | 3 | 1a | 0 | 0 | none | No | Yes | 1376 | 3/3 |
| **48** | Cerebellum-predominant | pure MSA | F | 69 | 4:30 | 0 | 2 | 0 | 1 | 3 | N/a | 0 | 0 | none | Occasional | Not mentioned | 1246 | 3/3 |
| **49** | Cerebellum-predominant | pure MSA | F | 59 | 6:40 | 2 | 0 | 0 | 2 | 3 | 0 | 0 | 0 | none | No | Not mentioned | 1102 | 3/3 |
| **50** | Cerebellum-predominant | pure MSA | M | 61 | 5:45 | 3 | 0 | 0 | 2 | 3 | 0 | 0 | 0 | none | No | Not mentioned | 1383 | 3/3 |
| **51** | Cerebellum-predominant | mixed MSA+AD | F | 76 | 4:50 | 3 | 3 | 4 | 1 | 2 | 0 | 2 | 1 | N/a | No | Not mentioned | 1278 | 3/4 |
| **52** | Cerebellum-predominant | pure MSA | M | 57 | 5:45 | 3 | 1 | 0 | 2 | 3 | N/a | 0 | 0 | none | Occasional | Not mentioned | 1345 | 3/3 |
| **53** | Cerebellum-predominant | pure MSA | F | 66 | 5:10 | 4 | 0 | 0 | 1 | 2 | 0 | 2 | 1 | none | No | Not mentioned | 1200 | 3/3 |
| **54** | Cerebellum-predominant | pure MSA | F | 62 | 5:35 | 4 | 0 | 0 | 2 | 3 | N/a | 0 | 0 | none | No | Not mentioned | 1050 | 2/3 |
| **55** | Cerebellum-predominant | pure MSA | F | 69 | 8:00 | 3 | 1 | 2 | 1 | 3 | 0 | 0 | 0 | none | No | Not mentioned | 1265 | 3/3 |
| **56** | Cerebellum-predominant | mixed MSA+AD | M | 74 | 3:50 | 3 | 3 | 4 | 2 | 3 | 0 | 2 | 1 | sparse | Occasional | Not mentioned | 1350 | 3/3 |
| **57** | Cerebellum-predominant | pure MSA | M | 70 | 6:55 | 3 | 1 | 1 | 1 | 3 | 0 | 2 | 2 | none | No | Not mentioned | 1325 | 3/3 |
| **58** | Cerebellum-predominant | pure MSA | F | 52 | 5:35 | 0 | 1 | 0 | 1 | 3 | 0 | 0 | 0 | none | No | Not mentioned | 1095 | 3/3 |
| **59** | Cerebellum-predominant | mixed MSA+AD | M | 70 | 4:05 | 4 | 5 | 4 | 1 | 2 | 0 | 1 | 3 | frequent | Occasional | Not mentioned | 1259 | 4/4 |
| **60** | Cerebellum-predominant | pure MSA | F | 68 | 12:00 | 6 | 1 | 2 | 2 | 3 | 0 | 0 | 0 | none | No | Not mentioned | n.a. | 3/4 |
| **61** | Cerebellum-predominant | pure MSA | M | 71 | 30:39 | 5 | 2 | N/a | 2 | 3 | 2 | N/a | N/a | N/a | Severe | Not mentioned | n.a. | 3/3 |
| **62** | MSA-mix | pure MSA | M | 65 | 4:45 | 3 | 1 | 0 | 3 | 3 | 0 | 0 | 0 | none | No | Not mentioned | 1439 | 3/4 |
| **63** | MSA-mix | pure MSA | M | 73 | 7:10 | 3 | 2 | 0 | 3 | 3 | 0 | 0 | 0 | none | Occasional | Not mentioned | 1290 | 3/3 |
| **64** | MSA-mix | pure MSA | F | 70 | 6:45 | 5 | 1 | 0 | 3 | 3 | 0 | 0 | 0 | none | Occasional | Not mentioned | 1178 | 3/3 |
| **65** | MSA-mix | pure MSA | M | 55 | 8:00 | 5 | 0 | 0 | 2 | 2 | 0 | 0 | 0 | none | No | Not mentioned | 1535 | 3/3 |
| **66** | MSA-mix | pure MSA | F | 74 | 6:45 | 3 | 2 | 1 | 3 | 3 | 1b | 1 | 1 | none | Occasional | Not mentioned | 1114 | 3/3 |
| **67** | MSA-mix | pure MSA | F | 67 | 4:00 | 6 | 2 | 0 | 2 | 2 | 0 | 0 | 0 | none | Occasional | Not mentioned | 1185 | 2/3 |
| **68** | MSA-mix | pure MSA | M | 54 | 6:05 | 5 | 0 | 2 | 3 | 3 | 0 | 0 | 0 | none | No | Not mentioned | 1230 | 2/3 |
| **69** | MSA-mix | pure MSA | M | 48 | 4:55 | 4 | 0 | 0 | 2 | 3 | 0 | 2 | 1 | none | Occasional | Not mentioned | 1371 | 2/4 |
| **70** | MSA-mix | mixed MSA+AD | M | 81 | 8:30 | 3 | 3 | 3 | 3 | 3 | 0 | 2 | 1 | sparse | Occasional | Not mentioned | 1290 | 3/4 |
| Neuropathological characteristics as based on the assessment of an experienced neuropathologist (A.J.M.R.). N/a = not available. MSA-mix = no predominant striatum or cerebellum pathology. Pure MSA = NFT Braak and amyloid Thal stages of ≤ 2, Mixed MSA+AD = NFT Braak or amyloid Thal stages of ≥ 3. PMD = time between death and autopsy. AGD = argyrophilic grain disease (considered positive when mentioned by the neuropathologist in the pathology report) | | | | | | | | | | | | | | | | | | |

|  | | **2. Case summaries of clinical features of MSA cohort** | | | | | | | | | | | | | | | | | | | | | | | | | | |
| --- | --- | --- | --- | --- | --- | --- | --- | --- | --- | --- | --- | --- | --- | --- | --- | --- | --- | --- | --- | --- | --- | --- | --- | --- | --- | --- | --- | --- |
| **Donor** | **Cohort group** | | **Sex** | **Age at onset (y)** | **Age at death (y)** | **Disease duration (y)** | **Cause of death** | **Final antemortem diagnosis** | **Initial antemortem diagnosis** | **Hoehn and Yahr (est.)** | **Parkinsonism?** | Duration (y) | **Cerebellar sympt.?** | Duration (y) | **Autonomic dysf.?** | Duration (y) | **CDR score** | **CDR estimated?** | **Hallucinations?** | **Depressed mood?** | Duration (y) | **RBD?** | **Nurinsg home?** | Duration (y) | **Levodopa response?** | Levodopa use (y) | **DBS?** |  |
| **1** | mixed MSA+AD | | F | 80 | 84 | 4 | Unknown | PD | PD | 5 | Yes | 4 | N/a |  | Yes | 2 | 0 | Yes | No | No |  | N/a | Yes | 1 | Unknown | N/a | No |  |
| **2** | pure MSA | | F | 55 | 63 | 8 | Euthanasia | PD | PD | 5 | Yes | 8 | Yes | 6 | Yes | 8 | 0,5 | Yes | N/a | Yes | 8 | N/a | N/a |  | Minimal, levodopa continued | 5 | No |  |
| **3** | pure MSA | | M | 46 | 57 | 9 | Respiratory failure | MSA | Parkinsonism | 5 | Yes | 11 | Yes | 10 | Yes | 10 | 0 | Yes | N/a | Yes | 11 | N/a | Yes | 6 | Never used | 0 | No |  |
| **4** | pure MSA | | M | 52 | 55 | 3 | Aspiration pneumonia | MSA | Parkinsonism | 4 | Yes | 2 | Yes | 2 | Yes | 4 | 0 | Yes | N/a | Yes | 1 | N/a | N/a |  | Marked, levodopa continued | 3 | No |  |
| **5** | pure MSA | | F | 81 | 84 | 3 | Respiratory failure | MSA | PD | 5 | Yes | 3 | No |  | Yes | 2 | 0 | Yes | N/a | N/a |  | N/a | Yes | 3 | Unknown | N/a | No |  |
| **6** | pure MSA | | F | 63 | 72 | 9 | Unknown | PSP | Parkinsonism | 4 | Yes | 9 | Yes | 5 | Yes | 8 | 1,0 | Yes | N/a | Yes | 9 | N/a | Yes | 5 | Minimal, levodopa continued | 7 | No |  |
| **7** | pure MSA | | F | 61 | 71 | 10 | Peritonitis | PD | PD | 5 | Yes | 10 | Yes | 4 | Yes | 6 | 0,5 | Yes | Yes | Yes | 8 | N/a | Yes | 7 | Minimal, levodopa continued | 9 | No |  |
| **8** | pure MSA | | F | 58 | 66 | 8 | Sepsis | MSA | PD | 5 | Yes | 6 | No |  | Yes | 4 | 0 | Yes | N/a | N/a |  | N/a | N/a |  | Minimal, levodopa continued | 10 | No |  |
| **9** | pure MSA | | F | 54 | 61 | 7 | Euthanasia | MSA | PD | 5 | Yes | 7 | No |  | Yes | 1 | 0 | Yes | Yes | No |  | No | Yes | 4 | Marked, levodopa continued | 3 | No |  |
| **10** | pure MSA | | M | 63 | 66 | 3 | Respiratory failure | MSA | DLB | 4 | Yes | 3 | Yes | 1 | Yes | 7 | 1,0 | Yes | Yes | No |  | No | Yes | 2 | No, levodopa stopped | 1 | No |  |
| **11** | mixed MSA+AD | | M | 72 | 79 | 7 | Cardiac failure | PDD | Parkinsonism | 5 | Yes | 7 | No |  | Yes | 3 | 2,0 | Yes | N/a | Yes | 6 | N/a | Yes | 6 | Minimal, levodopa continued | 3 | No |  |
| **12** | pure MSA | | F | 53 | 57 | 4 | Euthanasia | PD | PD | 4 | Yes | 4 | Yes | 4 | Yes | 1 | 0 | No | N/a | Yes | 3 | N/a | N/a |  | No, levodopa stopped | 1.5 | No |  |
| **13** | mixed MSA+AD | | F | 59 | 67 | 8 | Aspiration pneumonia | MSA | PD | 5 | Yes | 8 | No |  | Yes | 3 | 0,5 | No | No | Yes | 3 | N/a | Yes | 7 | No, levodopa stopped | 1.5 | No |  |
| **14** | mixed MSA+AD | | M | 66 | 70 | 4 | Advanced MSA | MSA | PD | 5 | Yes | 6 | Yes | 1 | Yes | 4 | 0,5 | No | Yes | Yes | 20 | Yes | No |  | No, levodopa stopped | 1 | No |  |
| **15** | pure MSA | | F | 64 | 70 | 6 | Pneumonia | MSA | MSA | 5 | Yes | 6 | Yes | 3 | Yes | 4 | 0,5 | No | Yes | Yes | 6 | N/a | No |  | Marked, levodopa continued | 5 | No |  |
| **16** | pure MSA | | F | 45 | 61 | 16 | Sepsis | MSA | PD | 5 | Yes | 16 | Yes | 9 | Yes | 12 | 0,5 | No | N/a | N/a |  | N/a | N/a |  | Minimal, levodopa continued | 14 | No |  |
| **17** | pure MSA | | F | 53 | 61 | 8 | Euthanasia | PD | PD | 5 | Yes | 8 | Yes | 8 | Yes | 1 | 0,5 | No | N/a | Yes | 10 | N/a | N/a |  | Moderate, levodopa continued | 8 | No |  |
| **18** | mixed MSA+AD | | M | 74 | 77 | 3 | Pneumonia | MSA | PD | 4 | Yes | 3 | Yes | 1 | Yes | 5 | 1,0 | No | N/a | N/a |  | N/a | N/a |  | Moderate, levodopa continued | 2 | No |  |
| **19** | mixed MSA+AD | | F | 58 | 60 | 2 | Euthanasia | MSA | MS | 4 | Yes | 3 | Yes | 1 | Yes | 3 | 1,0 | No | N/a | Yes | 18 | No | Yes | 2 | Moderate, levodopa continued | 2 | No |  |
| **20** | pure MSA | | M | 54 | 59 | 5 | Aspiration pneumonia | MSA | PD | 5 | Yes | 5 | Yes | 1 | Yes | 6 | 0 | No | N/a | N/a |  | No | N/a |  | Minimal, levodopa continued | 5 | No |  |
| **21** | mixed MSA+AD | | F | 80 | 83 | 3 | Euthanasia | PSP | PD | 4 | Yes | 3 | Yes | 2 | Yes | 0 | 0 | No | N/a | N/a |  | N/a | N/a |  | Unknown | N/a | No |  |
| **22** | mixed MSA+AD | | F | 71 | 74 | 3 | Euthanasia | MSA | PD | 4 | Yes | 3 | Yes | 1 | Yes | 2 | 1,0 | Yes | N/a | Yes | 3 | N/a | N/a |  | Minimal, levodopa continued | 3 | No |  |
| **23** | pure MSA | | F | 62 | 65 | 3 | Euthanasia | MSA | MSA | 5 | Yes | 3 | N/a | 1 | Yes | 2 | 0 | Yes | N/a | N/a |  | Yes | N/a |  | Marked, levodopa continued | 2 | No |  |
| **24** | pure MSA | | F | 46 | 49 | 3 | Pneumonia | MSA | MSA | 5 | Yes | 2 | Yes | 1 | Yes | 2 | 0 | No | N/a | N/a |  | N/a | Yes | 2 | No, levodopa continued | 2 | No |  |
| **25** | pure MSA | | F | 52 | 62 | 10 | Euthanasia | MSA | PD | 5 | Yes | 10 | No |  | Yes | 9 | 0,5 | No | N/a | N/a |  | Yes | Yes | 5 | Minimal, levodopa continued | 10 | No |  |
| **26** | pure MSA | | F | 56 | 59 | 3 | Pneumonia | MSA | PD | 5 | Yes | 3 | Yes | 3 | Yes | 2 | 0 | Yes | N/a | Yes | 3 | N/a | Yes | 3 | Minimal, levodopa continued | 3 | No |  |
| **27** | pure MSA | | F | 55 | 62 | 7 | Palliative sedation | MSA | PD | 5 | Yes | 7 | Yes | 6 | Yes | 6 | 0,5 | No | N/a | Yes | 5 | Yes | N/a |  | Minimal, levodopa continued | 7 | No |  |
| **28** | pure MSA | | F | 70 | 74 | 4 | Deceased during sleep | MSA | PD | 4 | Yes | 4 | Yes | 4 | Yes | 3 | 0,5 | Yes | Yes | Yes | 2 | Yes | No |  | Moderate, levodopa continued | 2 | No |  |
| **29** | pure MSA | | F | 67 | 72 | 5 | Unknown | MSA | PD | 5 | Yes | 5 | No |  | Yes | 2 | 0 | No | N/a | Yes |  | Yes | No |  | Minimal, levodopa continued | 5 | No |  |
| **30** | pure MSA | | M | 62 | 70 | 8 | Pneumonia | PD | PD | 5 | Yes | 8 | No |  | Yes | 6 | 0 | Yes | No | Yes | 14 | N/a | Yes | 7 | Marked, levodopa continued | 7 | Yes |  |
| **31** | mixed MSA+AD | | M | 80 | 84 | 4 | Euthanasia | PD | PD | 4 | Yes | 4 | Yes | 1 | Yes | 1 | 0 | No | N/a | Yes | 1 | N/a | No |  | Marked, levodopa continued | 4 | No |  |
| **32** | pure MSA | | M | 43 | 61 | 18 | Aspiration | MSA | PD | 5 | Yes | 19 | Yes | 4 | Yes | 7 | ,0 | Yes | N/a | No |  | Yes | Yes | 17 | Unknown | 19 | No |  |
| **33** | pure MSA | | F | 53 | 60 | 7 | Palliative sedation | MSA | PD | 5 | Yes | 7 | No |  | Yes | 4 | 0 | Yes | N/a | Yes | 3 | N/a | yes | 6 | Minimal, levodopa stopped | 4 | No |  |
| **34** | pure MSA | | F | 53 | 60 | 7 | Euthanasia | MSA | PD | 5 | Yes | 7 | Yes | 5 | Yes | 4 | 0,5 | No | N/a | Yes | 1 | N/a | N/a |  | Marked, levodopa continued | 4 | No |  |
| **35** | pure MSA | | M | 67 | 72 | 5 | Euthanasia | MSA | PD | 5 | Yes | 5 | Yes | 4 | Yes | 7 | 0 | No | No | No |  | No | N/a |  | Marked, levodopa continued | 5 | No |  |
| **36** | pure MSA | | F | 66 | 69 | 3 | Euthanasia | MSA | MSA | 5 | Yes | 3 | Yes | 2 | N/a |  | 0 | No | No | No |  | N/a | No |  | Unknown, levodopa stopped | 1 | No |  |
| **37** | pure MSA | | F | 73 | 74 | 1 | Euthanasia | MSA | MSA | 4 | Yes | 1 | Yes | 1 | Yes | 2 | 0,5 | Yes | Yes | No |  | No | No |  | Minimal, levodopa continued | 1 | No |  |
| **38** | pure MSA | | M | 61 | 68 | 7 | Pneumonia | MSA | PD | 5 | Yes | 7 | Yes | 3 | Yes | 3 | 0,5 | No | No | Yes | 6 | Yes | No |  | Moderate, levodopa continued | 7 | No |  |
| **39** | pure MSA | | F | 59 | 65 | 6 | Respiratory failure | MSA | PD | 5 | Yes | 6 | Yes | 2 | Yes | 4 | 0 | No | Yes | No |  | Yes | No |  | Minimal, levodopa continued | 7 | No |  |
| **40** | mixed MSA+AD | | F | 58 | 67 | 9 | Euthanasia | PD | PD | 5 | Yes | 4 | Yes | 4 | Yes | 3 | 0,5 | Yes | N/a | Yes | 22 | N/a | No |  | Marked, levodopa continued | 4 | No |  |
| **41** | pure MSA | | F | 48 | 52 | 4 | Euthanasia | MSA | PTSD | 4 | Yes | 4 | Yes | 1 | Yes | 4 | 0,5 | Yes | N/a | Yes | 1 | Yes | No |  | Minimal, levodopa continued | 1 | No |  |
| **42** | pure MSA | | M | 49 | 54 | 5 | Collapse | MSA | MSA | 5 | Yes | 5 | No |  | Yes | 7 | 0 | Yes | No | No |  | Yes | No |  | No, levodopa stopped | N/a | No |  |
| **43** | pure MSA | | M | 51 | 60 | 9 | Sepsis | MSA | MSA | 5 | Yes | 9 | N/a |  | Yes | 6 | 1,0 | No | N/a | Yes |  | N/a | Yes |  | Moderate, levodopa continued | N/a | No |  |
| **44** | pure MSA | | F | 51 | 57 | 6 | Pneumonia | MSA | MSA | 5 | Yes | 6 | N/a |  | Yes | 6 | 0 | Yes | N/a | N/a |  | N/a | Yes | 5 | Unknown, levodopa continued | 4 | No |  |
| **45** | pure MSA | | F | 50 | 59 | 9 | Suicide | MSA | PD | 5 | Yes | 9 | Yes | 9 | Yes | 4 | 0,5 | Yes | Yes | Yes | 7 | Yes | No |  | Minimal, levodopa continued | 9 | Yes |  |
| **46** | pure MSA | | M | 57 | 66 | 9 | Sepsis | MSA | MSA | 5 | No |  | Yes | 9 | Yes | 5 | 0 | Yes | N/a | N/a |  | N/a | No |  | No, levodopa stopped | 0.2 | No |  |
| **47** | pure MSA | | M | 65 | 67 | 2 | Aspiration pneumonia | MSA | MSA | 5 | Yes | 1 | Yes | 2 | Yes | 5 | 0 | No | N/a | Yes | 1 | N/a | Yes | 2 | No, levodopa stopped | 0.2 | No |  |
| **48** | pure MSA | | F | 63 | 69 | 6 | Euthanasia | MSA | MSA | 5 | No |  | Yes | 4 | Yes | 6 | 0 | No | N/a | Yes | 5 | N/a | N/a |  | No, levodopa stopped | 0.4 | No |  |
| **49** | pure MSA | | F | 54 | 59 | 5 | Euthanasia | MSA | PD | 4 | Yes | 5 | Yes | 5 | Yes | 4 | 0,5 | No | Yes | Yes | 1 | Yes | N/a |  | Minimal, levodopa continued | 2 | No |  |
| **50** | pure MSA | | M | 56 | 61 | 5 | Euthanasia | MSA | MSA | 5 | Yes | 2 | Yes | 5 | Yes | 6 | 0 | No | N/a | No |  | N/a | N/a |  | Never used | 0 | No |  |
| **51** | mixed MSA+AD | | F | 75 | 76 | 1 | Euthanasia | AD | AD | 5 | No |  | Yes | 1 | N/a |  | 2,0 | Yes | N/a | Yes | 4 | N/a | No |  | Never used | 0 | No |  |
| **52** | pure MSA | | M | 53 | 57 | 4 | Euthanasia | MSA | MSA | 5 | Yes | 3 | Yes | 4 | Yes | 3 | 0 | No | No | No |  | Yes | No |  | Never used | 0 | No |  |
| **53** | pure MSA | | F | 61 | 66 | 5 | Euthanasia | MSA | MSA | 5 | Yes | 3 | Yes | 5 | Yes | 3 | 0 | No | No | Yes | 2 | Yes | N/a |  | Unknown | N/a | No |  |
| **54** | pure MSA | | F | 58 | 62 | 4 | Euthanasia | MSA | Cerebellar syndrome | 5 | Yes | 1 | Yes | 4 | Yes | 4 | 0 | No | N/a | No |  | N/a | N/a |  | Never used | 0 | No |  |
| **55** | pure MSA | | F | 67 | 69 | 2 | Euthanasia | MSA | MSA | 4 | No |  | Yes | 2 | N/a |  | 0,5 | No | N/a | N/a |  | No | N/a |  | Never used | 0 | No |  |
| **56** | mixed MSA+AD | | M | 67 | 74 | 7 | Pneumonia | MSA | Cerebellar syndrome | 5 | Yes | 5 | Yes | 7 | Yes | 4 | 1,0 | Yes | No | No |  | Yes | Yes | 6 | No, levodopa stopped | N/a | No |  |
| **57** | pure MSA | | M | 62 | 70 | 8 | Euthanasia | Cerebellar syndrome | Cerebellar syndrome | 5 | Yes | 1 | Yes | 8 | No |  | 0,5 | No | N/a | Yes | 4 | N/a | Yes | 7 | Unknown | N/a | No |  |
| **58** | pure MSA | | F | 49 | 52 | 3 | Euthanasia | MSA | MSA | 5 | Yes | 1 | Yes | 3 | Yes | 3 | 0 | No | N/a | Yes | 6 | Yes | Yes | 3 | Never used | 0 | No |  |
| **59** | mixed MSA+AD | | M | 66 | 70 | 4 | End stage dementia | MSA | MSA | 4 | Yes | 4 | Yes | 4 | Yes | 4 | 2,0 | Yes | No | Yes | 3 | Yes | Yes | 4 | Never used | 0 | No |  |
| **60** | pure MSA | | F | 66 | 68 | 2 | Deceased during sleep | MSA | MSA | 4 | No |  | Yes | 2 | Yes | 3 | 0 | Yes | No | N/a |  | N/a | N/a |  | Unknown | N/a | No |  |
| **61** | pure MSA | | M | 71 | 71 | 1 | Unknown | MSA | MSA | 5 | N/a |  | N/a |  | N/a |  | 0 | No | N/a | N/a |  | N/a | N/a |  | No | N/a | No |  |
| **62** | pure MSA | | M | 61 | 65 | 4 | Unknown | MSA | MSA | 4 | Yes | 4 | N/a |  | Yes | 2 | 0,5 | Yes | N/a | Yes | 4 | N/a | N/a |  | Unknown | N/a | No |  |
| **63** | pure MSA | | M | 66 | 73 | 7 | Euthanasia | MSA | MSA | 5 | No |  | Yes | 5 | Yes | 8 | 0 | No | N/a | N/a |  | N/a | N/a |  | Never used | 0 | No |  |
| **64** | pure MSA | | F | 65 | 70 | 5 | Euthanasia | MSA | MSA | 4 | Yes | 2 | Yes | 5 | Yes | 5 | 0 | No | N/a | Yes | 13 | N/a | N/a |  | Unknown, levodopa continued | 2 | No |  |
| **65** | pure MSA | | M | 50 | 55 | 5 | Advanced MSA | MSA | MSA | 5 | No |  | Yes | 5 | Yes | 4 | 0 | Yes | N/a | No |  | Yes | No |  | Never used | 0 | No |  |
| **66** | pure MSA | | F | 69 | 74 | 5 | Respiratory failure | MSA | PD | 5 | Yes | 5 | Yes | 4 | Yes | 14 | 0,5 | No | N/a | Yes | 2 | N/a | Yes | 3 | Minimal, levodopa stopped | 2 | No |  |
| **67** | pure MSA | | F | 62 | 67 | 5 | Dehydration | MSA | Parkinsonism | 5 | Yes | 5 | Yes | 2 | Yes | 2 | 0 | No | No | No |  | No | Yes | 5 | Marked, levodopa continued | 5 | No |  |
| **68** | pure MSA | | M | 45 | 54 | 9 | Euthanasia | MSA | PD | 4 | Yes | 9 | Yes | 6 | Yes | 7 | 1,0 | No | N/a | N/a |  | Yes | No |  | Moderate, levodopa continued | 9 | No |  |
| **69** | pure MSA | | M | 41 | 48 | 7 | Euthanasia | MSA | Parkinsonism | 5 | Yes | 7 | Yes | 4 | Yes | 7 | 0 | No | No | Yes | 7 | Yes | No |  | Marked, levodopa continued | 9 | Yes |  |
| **70** | mixed MSA+AD | | M | 74 | 81 | 7 | Aspiration | MSA | MSA | 4 | Yes | 5 | Yes | 7 | Yes | 6 | 0 | Yes | N/a | N/a |  | Yes | No |  | Unknown | N/a | No |  |
|  | | All clinical symptoms were based on clinical reports of treating physician. N/a = not available. Hoehn and Yahr scores were estimated based on physician’s clinical reports in year prior to death. Duration gives information on years between onset and death of symptom stated in column before. Parkinsonism = bradykinesia with tremor and/or rigidity. Cerebellar symptoms = ataxia, cerebellar dysarthria. Autonomic dysfunction = urinary incontinence or orthostatic hypotension. REM behavioral sleep disorder (RBD) = recorded as present if confirmed on polysomnography or if it was clinically suspected based on the behavioral description by the bed partner. Nursing home = permanent stay in nursing home. Levodopa response = as reported by patient and physician and subsequently categorized as never used/no/minimal/moderate/marked/significant. DBS = deep brain stimulation | | | | | | | | | | | | | | | | | | | | | | | | | |  |

**3. Staining protocols immunohistochemistry**

*Immunohistochemistry staining protocol*

Staining protocols for α-syn (KM51), Aβ (6F/3D) and p-tau (AT8) were previously published [10]. For the pTDP-43 (pSer409) staining, after deparaffinization and rinsing in TBS pH 9.0, antigen retrieval was performed for 10 minutes in TBS (pH 9.0) in a microwave oven. Consequently, the slides were rinsed in PBS, and a second antigen retrieval step was performed with 1% formic acid and PBS. The sections were incubated in anti-TDP43 overnight at 4ºC. Afterwards, the slides were incubated with the secondary antibody from the Envision-kit for 1 hour and DAB chromogen was consecutively applied to the slides for 10 minutes. Ultimately, a haematoxylin staining was performed for 2 minutes, sections were mounted using Entellan (*Sigma-Aldrich*) and a coverslip was applied to all sections.

| **Summary of antibodies used in immunohistochemistry** | | | | | |
| --- | --- | --- | --- | --- | --- |
| **Primary antibody** | **Clone** | **Antibody ID** | **Source** | **Dilution** | **Host** |
| Alpha-synuclein | KM51 | NCL-L-ASYN; MONX10739 | Novocastra, Leica AND Monosan Xtra, The Netherlands | 1/500 | Mouse |
| Amyloid-beta | 6F/3D | M087201 | DAKO, Denmark | 1/500 | Mouse |
| Phosphorylated-tau | AT8 | MN1020 | Thermo Fisher Scientific, USA | 1/500 | Mouse |
| pTDP-43 | Phospho ps409/410 | CAC-TIP-PTD-P03 | Cosmo Bio, USA | 1/10.000 | Mouse |

**4. Regional segmentations of the amygdala and hippocampus**

Different outcome measures were used to best measure pathological burden in each brain region. In-house developed scripts, as published previously [10], were used to measure total immunopositivity in each brain region. To obtain %area, the entire region of interest (amygdala, CA1, CA2, CA3+4, DG and EntC) was used. Since manual characterization of pathological structures based on their morphology was quite labor intensive to perform for the entire region, squares for quantification were placed within the amygdala (all pathologies) and EntC (only for α-syn) as described in the table below.

| **Brain region** | **Area for manual morphological distinction** | **Region of interest with their sub delineations** |
| --- | --- | --- |
| Amygdala  – for all pathological stainings | 8 squares of 1 mm^2^ – randomly placed by QuPath after subdividing amygdala in 4 quarters | 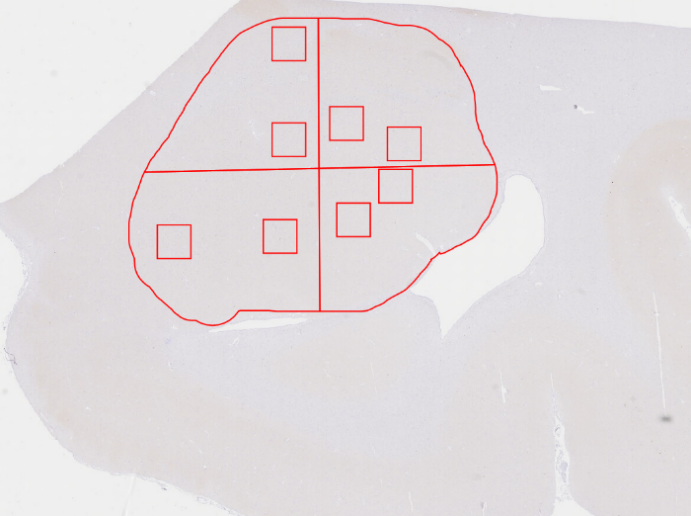 |
| Hippocampus:  CA1 – CA2 – CA3+4 – DG  – for all pathological stainings | Complete region of interest | 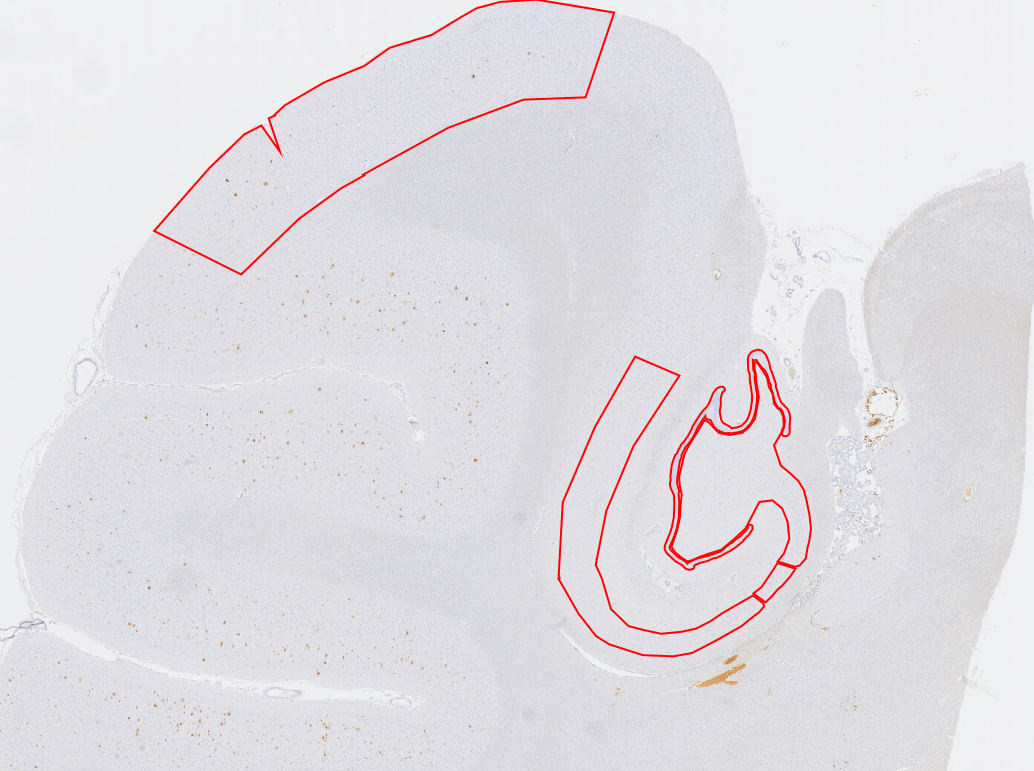 |
| Entorhinal Cortex  – alpha-synuclein | 4 squares of 1 mm^2^ – randomly placed by Qupath | 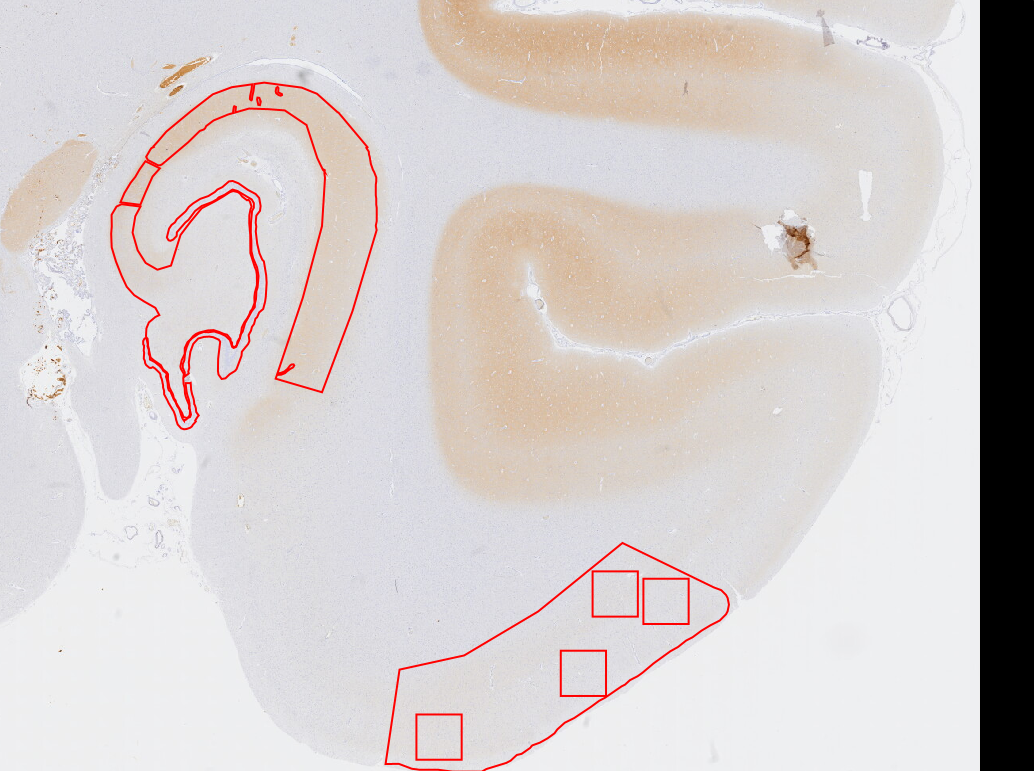 |
| Entorhinal Cortex  – amyloid-beta and p-Tau | Complete region of interest | 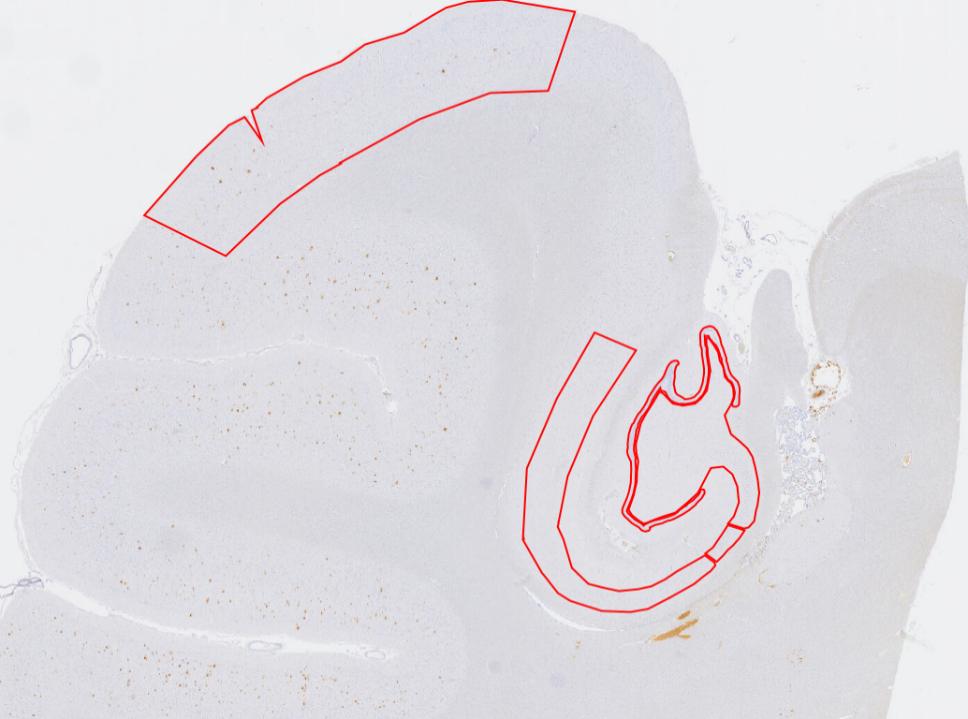 |

**5. Detection of antibody immunopositivity by in-house developed QuPath scripts**


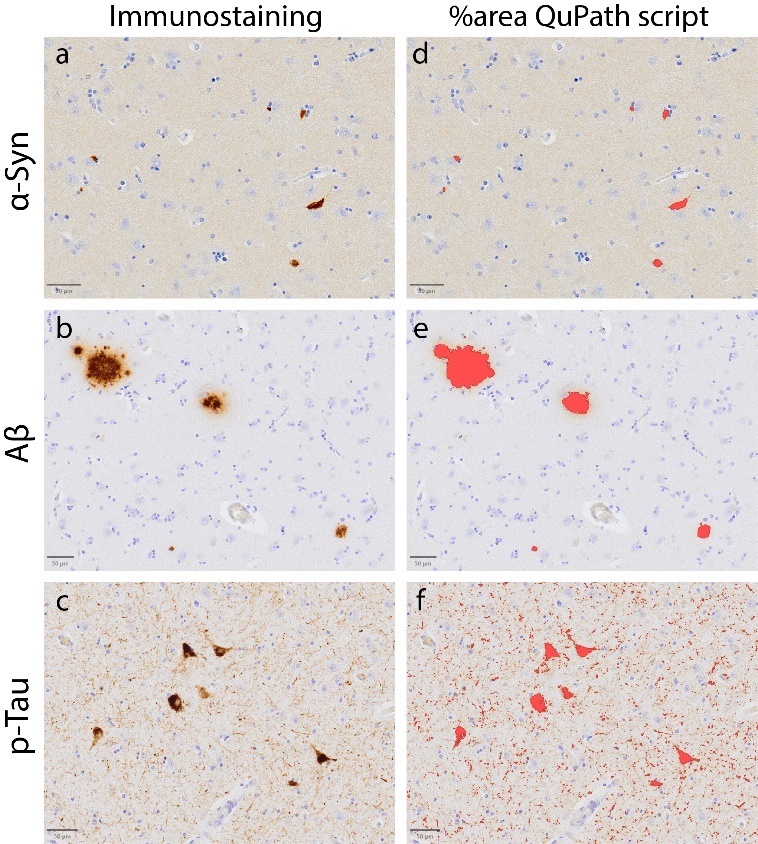
**a** Immunostaining against full-length alpha-synuclein (KM51), demonstrating oligodendroglial, neuronal and neuritic alpha-synuclein positivity. **b** Immunostaining against amyloid-beta (6F/3D) demonstrating diffuse neuritic amyloid-beta plaques. **c** Immunostaining against p-tau (AT8) showed neurofibrillary tangles, neuropil threads and accumulated p-tau. **d-f** Shown in red, specific DAB staining identified as ‘positive’ by the respective QuPath script, based on pixel quantification

**
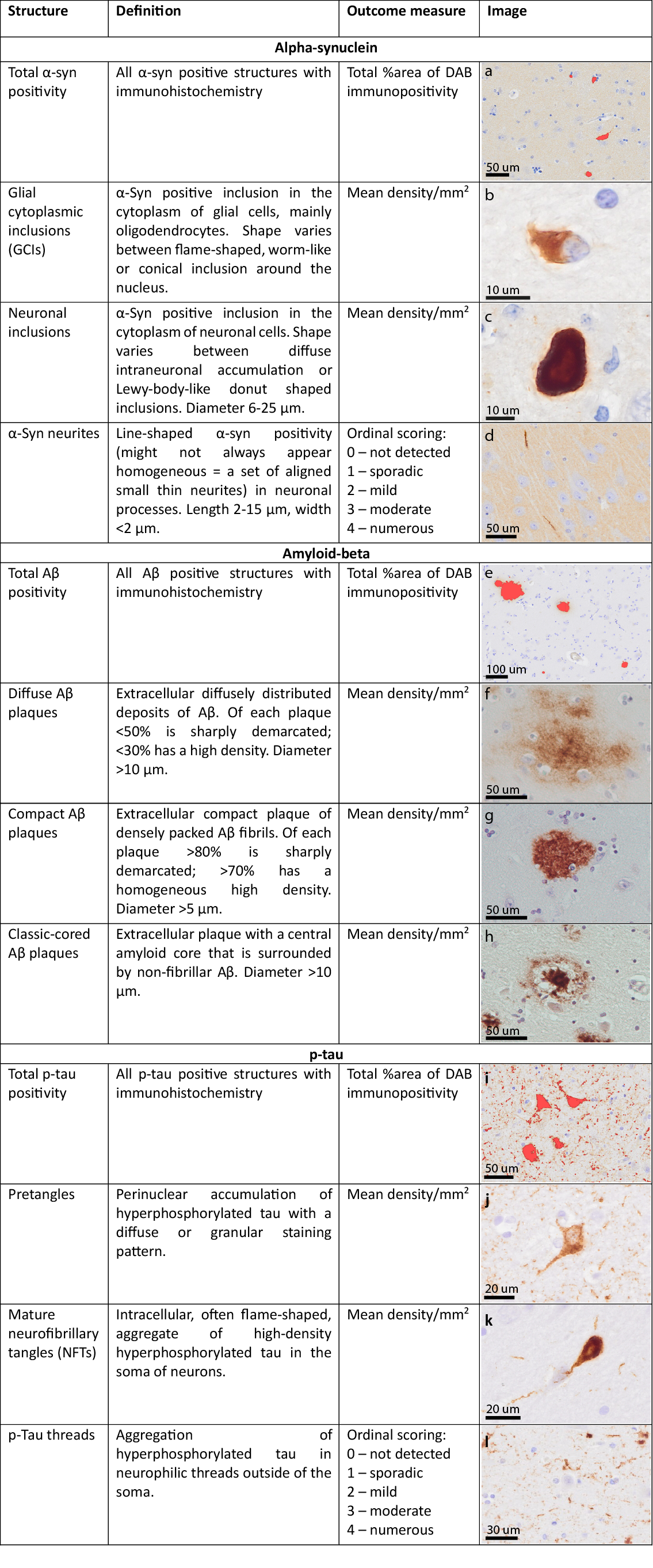
6. Outcome measures of pathology detection**

Different outcome measures were used to best describe pathological burden in brain regions. See definitions and representative images for α-syn, Aβ and p-tau inclusions here. Images were made in the amygdala for image **a-d, f, g, h, i**, in CA for image **e** and in EntC for image **j, k, l**. Scale bars differ per image as described within the image

**7. Demographics of cohort based on pathological MSA subtypes (striatum- vs. cerebellum-predominant)**

As described, no differences were observed in co-pathology burden between pathological striatum-predominant MSA, cerebellum-predominant MSA or MSA-mix (striatum = cerebellum). See the characteristics of these pathological subgroups below.

| **Table 1. Group demographics, neuropathological stages and genotype of pathological MSA subtypes** | | | | | | | | |  |
| --- | --- | --- | --- | --- | --- | --- | --- | --- | --- |
|  | **Striatum-predominant**  **MSA** | | **Cerebellum-predominant**  **MSA** | | | **MSA-mix  (striatum = cerebellum)** | | ***P*-value** ^1^ | |
| ***N*** | 43 | | 18 | | | 9 | |  | |
| **Sex** M/F (%M) | 14/29 (33%) | | 8/10 (44%) | | | 6/3 (67%) | | n.s. | |
| **Age at death** y mean ± SD (range) | 66 ± 9 | | 65 ± 7 | | | 65 ± 11 | | n.s. | |
| **Co-pathology subtype (%)**  **Pure MSA-AD** N (%)  **Mixed MSA+AD** N (%) | 33 (77%)  10 (23%) | | 15 (83%)  3 (17%) | | | 8 (89%)  1 (11%) | | n.s. | |
| **Braak α-syn [1]** 0/1/2/3/4/5/6 | 11/0/0/5/7/12/8 | | 4/0/1/8/3/1/1 | | | 0/0/0/4/1/3/1 | | *p* = 0.029 | |
| **MSA-SND stage [4]** 0/1/2/3 | 0/0/5/38 | | 0/10/8/0 | | | 0/0/3/6 | | *p* < 0.001 | |
| **MSA-OPCA stage [4]** 0/1/2/3 | 3/26/14/0 | | 0/0/4/14 | | | 0/0/2/7 | | *p* < 0.001 | |
| **Thal** **Aβ [3]** 0/1/2/3/4/5 | 17/12/7/3/4/0 | | 10/1/2/0/3/0 | | | 6/1/1/1/0/0 | | n.s. | |
| **Braak NFT [2]** 0/1/2/3/4/5/6 | 11/19/7/3/3/0/0 | | 7/6/2/2/0/1/0 | | | 3/2/3/1/0/0/0 | | n.s. | |
| **LATE-NC TDP^1^ [5]** 0/1a/1b/2/3 | 35/4/0/1/0 | | 12/1/0/1/0 | | | 8/0/1/0/0 | | n.s. | |
| **CAA stage [11]** 0/1/2/3 | 27/10/4/1 | | 11/3/1/1 | | | 6/3/0/0 | | n.s. | |
| **CERAD [7]** 0/1/2/3 | 31/9/0/1 | | 12/1/0/1 | | | 8/1/0/0 | | n.s. | |
| **APOE-ε4 alleles**  Non-carrier/hetero-/homozygous | 24/15/4 | | 13/4/1 | | | 6/3/0 | | n.s. | |
| N.s. = no significant difference between groups. SND = striatonigral degeneration; OPCA = olivopontocerebellar atrophy; LATE-NC = Limbic-predominant age-related TDP-43 encephalopathy; CAA = cerebral amyloid angiopathy; CERAD = Consortium to Establish a Registry for Alzheimer Disease; APOEε = apolipoprotein E. Groups were compared using a one-way ANOVA and a Fisher’s exact test for categorical variables. ^1^LATE-NC staging was missing for 7 donors | | | | | | | | | |
| **Table 2. Clinical features of pathological MSA subtypes** | | | | | | | | |  |
|  | | **Striatum-predominant**  **MSA** | | **Cerebellum-predominant**  **MSA** | | | **MSA-mix  (striatum = cerebellum)** | | ***P*-value** ^1^ |
| ***N*** | | 43 | | 18 | | | 9 | |  |
| **Sex** M/F (%M) | | 14/29 (33%) | | 8/10 (44%) | | | 6/3 (67%) | | n.s. |
| **Age at onset** y mean ± SD (range) | | 61 ± 10 | | 61 ± 7 | | | 59 ± 11 | | n.s. |
| **Age at death** y mean ± SD (range) | | 66 ± 9 | | 65 ± 7 | | | 65 ± 11 | | n.s. |
| **Disease duration** y mean ± SD (range) | | 6 ± 4 | | 5 ± 2 | | | 6 ± 2 | | n.s. |
| **Final antemortem diagnosis** MSA/not MSA (%matching post-mortem) | | 32/11 (74%) | | 16/2 (89%) | | | 9/0 (100%) | | n.s. |
| **Final antemortem diagnosis specified**  MSA-P/MSA-C/MSA-unspecified/ PD/PDD/PSP/AD/cerebellar atrophy | | 22/0/10/  8/1/2/0/0 | | 0/9/7/  0/0/0/1/1 | | | 2/4/3/  0/0/0/0/0 | | *p* < 0.001 |
| **Initial antemortem diagnosis specified**  MSA-unspecified/PD/DLB/parkinsonism/  AD/cerebellar syndrome/MS/PTSD | | 7/29/1/4/  0/0/1/1 | | 12/2/0/0/  1/3/0/0 | | | 5/2/0/2/  0/0/0/0 | | *p* < 0.001 |
| **Hoehn and Yahr estimated** 0/1/2/3/4/5 | | 0/0/0/0/12/31 | | 0/0/0/0/4/14 | | | 0/0/0/0/4/5 | | n.s. |
| **Clinical Dementia Rating scale (CDR)** 0/0.5/1/2 | | 21/15/6/1 | | 11/4/1/2 | | | 6/2/1/0 | | n.s. |
| **Parkinsonism** yes/no (%yes) | | 43/0 (100%) | | | 13/5 (72%) | | 7/2 (78%) | | *p* < 0.001 |
| **Cerebellar symptoms** yes/no (%yes) | | 30/10 (75%) | | | 17/0 (100%) | | 8/0 (100%) | | n.s. |
| **Autonomic dysfunction** yes/no (%yes) | | 42/0 (100%) | | | 15/1 (94%) | | 9/0 (100%) | | n.s. |
| **Hallucinations** yes/no (%yes) | | 8/7 (53%) | | | 3/5 (63%) | | 0/2 (0%) | | n.s. |
| **Depressed mood** yes/no (%yes) | | 25/9 (74%) | | | 10/4 (71%) | | 4/2 (67%) | | n.s. |
| **RBD** yes/no (%yes) | | 11/6 (65%) | | | 7/2 (78%) | | 4/1 (80%) | | n.s. |
| **Levodopa response** yes/no (%yes) | | 21/13 (62%) | | | 2/5 (29%) | | 2/2 (50%) | | n.s. |
| Clinical characteristics were only reported if specifically stated as present or not present in clinical reports, not all information was available. N.s. = no significant difference between groups. CDR = clinical dementia rating score; RBD = REM behavioral sleep disorder. Groups were compared using a one-way ANOVA for continuous and a Fisher’s exact test for categorical variables | | | | | | | | | |

**8. Pairwise comparisons of pathological burden between pure MSA and mixed MSA+AD**

|  | **EntC** | **Amy** | **CA1** | **CA2** | **CA3+4** | **DG** |
| --- | --- | --- | --- | --- | --- | --- |
| **α-syn %area** | -0.28  (0.680) | -0.39  (0.488) | -1.51  (0.584) | -1.91  (0.623) | -0.41  (0.718) | 0.00  (0.999) |
| **α-syn GCIs** | 1.95  (0.513) | 0.550  (0.942) | 1.44  (0.492) | 5.22  (0.330) | 0.642  (0.543) | 0.44  (0.406) |
| **α-syn neuronal inclusions** | 0.17  (0.490) | -2.08  (0.199) | 0.37  (0.597) | 2.83  (0.226) | 0.25  (0.534) | 1.49  (0.610) |
| **α-syn neurites** | 0.22  (0.489) | **-1.03**  **(0.012)** | 0.60  (0.127) | 0.25  (0.505) | 0.63  (0.059) | 0.44  (0.100) |
| **Aβ %area** | **-1.61**  **(0.002)** | **-0.47**  **(0.007)** | **-0.36 (0.004)** | **-0.13**  **(0.022)** | -0.13  (0.058) | -0.17  (0.069) |
| **Aβ diffuse plaques** | **-17.40**  **(<0.001)** | **-5.14**  **(0.014)** | **-1.59**  **(0.014)** | -3.29  (0.133) | **-0.70**  **(0.018)** | **-1.54**  **(0.001)** |
| **Aβ compact plaques** | **-2.12**  **(0.008)** | **-2.26**  **(0.028)** | **-0.66**  **(0.024)** | **-0.44**  **(0.008)** | **-0.34**  **(0.029)** | -0.54  (0.167) |
| **Aβ classic-cored plaques** | **-0.44**  **(<0.001)** | **-0.16**  **(0.009)** | **-0.06**  **(<0.001)** | n/a | n/a | n/a |
| **p-Tau %area** | -0.75  (0.054) | **-6.54**  **(0.011)** | **-2.34**  **(0.017)** | **-5.54**  **(0.046)** | -1.37  (0.082) | **-0.44**  **(0.037)** |
| **p-Tau pretangles** | **-1.08**  **(0.005)** | -0.41  (0.668) | **-5.01**  **(0.006)** | -3.88  (0.094) | **-0.46**  **(0.019)** | **-5.24**  **(0.027)** |
| **p-Tau NFTs** | **-2.03**  **(0.008)** | -1.25  (0.184) | **-8.66**  **(0.002)** | **-16.04**  **(0.004)** | **-3.26**  **(0.007)** | -0.85  (0.167) |
| **p-Tau threads** | -0.64  (0.109) | **-1.36**  **(<0.001)** | **-1.12**  **(<0.001)** | **-1.00**  **(0.006)** | **-1.12**  **(<0.001)** | **-1.27**  **(<0.001)** |
| Pairwise comparisons on pathological burden between pure MSA and mixed MSA+AD donors across limbic regions. Mean difference and *p*-value (between brackets) are depicted below each other. Significant *p*-values are demonstrated in **bold**. Groups were compared using a univariate ANCOVA adjusted for age at death and sex, and corrected for multiple comparisons using Bonferroni. n/a = not applicable; pathology was not present in this brain region | | | | | | |

**9. Pairwise comparisons of pathological burden across limbic regions**

|  |  | **α-syn %area** | **α-syn GCIs** | **α-syn neuronal inclusions** | **α-syn neurites** | **Aβ %area** | **Aβ diffuse plaques** | **Aβ compact plaques** | **Aβ classic-cored plaques** | **p-Tau %area** | **p-Tau pretangles** | **p-Tau NFTs** | **p-Tau threads** |
| --- | --- | --- | --- | --- | --- | --- | --- | --- | --- | --- | --- | --- | --- |
| **EntC** | **Amygdala** | 0.03 | -4.64 | -1.65 | **-0.66** | 0.22 | 2.81 | -0.44 | 0.07 | -1.06 | -0.35 | 0.01 | 0.48 |
|  |  | (1.000) | (0.209) | (0.583) | **(0.002)** | (1.000) | (0.250) | (1.000) | (0.146) | (1.000) | (1.000) | (1.000) | 0.074 |
|  | **CA1** | -0.74 | **5.98** | -0.49 | -0.24 | 0.28 | **4.88** | 0.26 | **0.10** | -0.31 | -2.00 | -2.40 | 0.26 |
|  |  | (1.000) | **(0.021)** | (1.000) | (1.000) | (0.327) | **(<0.001)** | (1.000) | **(0.002)** | (1.000) | (0.206) | (1.000) | (1.000) |
|  | **CA2** | -2.25 | 0.17 | **-2.67** | 0.07 | 0.32 | **4.35** | 0.29 | **0.11** | -1.18 | **-3.28** | **-5.03** | 0.40 |
|  |  | (0.257) | (1.000) | **(0.012)** | (1.000) | (0.115) | **(0.003)** | (1.000) | **(<0.001)** | (1.000) | **(<0.001)** | **(0.004)** | 0.279 |
|  | **CA3+4** | -0.15 | **5.87** | -0.09 | 0.16 | 0.32 | **5.20** | 0.33 | **0.11** | 0.02 | 0.54 | 0.17 | **0.86** |
|  |  | (1.000) | **(0.028)** | (1.000) | (1.000) | (0.131) | **(<0.001)** | (1.000) | **(<0.001)** | (1.000) | (1.000) | (1.000) | **(<0.001)** |
|  | **DG** | 0.37 | **8.00** | **-3.72** | 0.42 | 0.31 | **4.98** | 0.29 | **0.11** | 0.23 | -1.17 | 0.75 | **1.44** |
|  |  | (1.000) | **(<0.001)** | **(<0.001)** | (0.182) | (0.139) | **(<0.001)** | (1.000) | **(<0.001)** | (1.000) | (1.000) | (1.000) | **(<0.001)** |
| **Amygdala** | **CA1** | -0.76 | **10.62** | 1.15 | 0.42 | 0.05 | 2.07 | 0.71 | 0.03 | 0.75 | -1.65 | -2.41 | -0.22 |
|  |  | (1.000) | **(<0.001)** | (1.000) | (0.200) | (1.000) | (1.000) | (0.194) | (1.000) | (1.000) | (0.643) | (1.000) | 1.000 |
|  | **CA2** | -2.28 | 4.81 | -1.02 | **0.73** | 0.10 | 1.55 | 0.73 | 0.05 | -0.12 | **-2.93** | **-5.04** | -0.08 |
|  |  | (0.224) | (0.149) | (1.000) | **(<0.001)** | (1.000) | (1.000) | (0.150) | (1.000) | (1.000) | **(0.005)** | **(0.004)** | 1.000 |
|  | **CA3+4** | -0.18 | **10.51** | 1.56 | **0.81** | 0.09 | 2.39 | 0.77 | 0.05 | 1.08 | 0.89 | 0.16 | 0.39 |
|  |  | (1.000) | **(<0.001)** | (0.734) | **(<0.001)** | (1.000) | (0.617) | (0.100) | (1.000) | (1.000) | (1.000) | (1.000) | 0.318 |
|  | **DG** | 0.34 | **12.64** | -2.08 | **1.08** | 0.08 | 2.17 | 0.73 | 0.05 | 1.29 | -0.82 | 0.74 | **0.97** |
|  |  | (1.000) | **(<0.001)** | (0.127) | **(<0.001)** | (1.000) | (0.953) | (0.151) | (1.000) | (1.000) | (1.000) | (1.000) | **(<0.001)** |
| **CA1** | **CA2** | -1.52 | **-5.81** | -2.18 | 0.31 | 0.04 | -0.53 | 0.03 | 0.01 | -0.87 | -1.28 | -2.63 | 0.14 |
|  |  | (1.000) | **(0.025)** | (0.082) | (0.893) | (1.000) | (1.000) | (1.000) | (1.000) | (1.000) | (1.000) | (0.808) | (1.000) |
|  | **CA3+4** | 0.58 | -0.11 | 0.41 | 0.40 | 0.04 | 0.32 | 0.07 | 0.01 | 0.33 | **2.54** | 2.57 | **0.61** |
|  |  | (1.000) | (1.000) | (1.000) | (0.259) | (1.000) | (1.000) | (1.000) | (1.000) | (1.000) | **(0.025)** | (0.906) | **(0.004)** |
|  | **DG** | 1.10 | 2.01 | **-3.23** | 0.67 | 0.03 | 0.10 | 0.03 | 0.01 | 0.54 | 0.83 | 3.15 | **1.19** |
|  |  | (1.000) | (1.000) | **(<0.001)** | (0.001) | (1.000) | (1.000) | (1.000) | (1.000) | (1.000) | (1.000) | (0.321) | **(<0.001)** |
| **CA2** | **CA3+4** | 2.10 | **5.70** | **2.58** | 0.08 | 0.00 | 0.84 | 0.04 | 0.00 | 1.20 | **3.81** | **5.20** | 0.47 |
|  |  | (0.366) | **(0.033)** | **(0.016)** | (1.000) | (1.000) | (1.000) | (1.000) | (1.000) | (1.000) | **(<0.001)** | **(0.002)** | (0.075) |
|  | **DG** | 2.62 | **7.83** | -1.05 | 0.35 | -0.01 | 0.62 | 0.00 | 0.00 | 1.41 | 2.11 | **5.78** | **1.05** |
|  |  | (0.076) | **(<0.001)** | (1.000) | (0.518) | (1.000) | (1.000) | (1.000) | (1.000) | (1.000) | (0.130) | **(<0.001)** | **(<0.001)** |
| **CA**  **3+4** | **DG** | 0.52 | 2.13 | **-3.64** | 0.27 | -0.01 | -0.22 | -0.04 | 0.00 | 0.21 | -1.71 | 0.58 | **0.58** |
|  |  | (1.000) | (1.000) | **(<0.001)** | (1.000) | (1.000) | (1.000) | (1.000) | (1.000) | (1.000) | (0.505) | (1.000) | **(0.009)** |

Pairwise comparisons on pathological burden between limbic regions. Mean difference and *p*-value (between brackets) are depicted below each other. Significant *p*-values are demonstrated in **bold**. Regions were compared using a univariate ANCOVA adjusted for age at death and sex, and corrected for multiple comparisons using Bonferroni. n/a = not applicable; pathology was not present in this brain region

**10. Associations between neuropathological burden, pathological stages and clinical measures**

| **a.**  **vs.** | **α-syn GCIs** | **α-syn neuronal inclusions** | **α-syn neurites** | **Aβ diffuse plaques** | **Aβ compact plaques** | **Aβ classic-cored plaques** | **p-tau pretangles** | | | **p-tau NFTs** | | | **p-tau threads** | |  |  |
| --- | --- | --- | --- | --- | --- | --- | --- | --- | --- | --- | --- | --- | --- | --- | --- | --- |
| **α-syn GCIs** | x | **0.26** | **0.33** | 0.05 | 0.03 | 0.07 | -0.03 | | | -0.02 | | | 0.08 | |  |  |
|  |  | **(0.000)** | **(0.000)** | (0.438) | (0.651) | (0.298) | (0.739) | | | (0.764) | | | (0.129) | |  |  |
| **α-syn neuronal inclusions** | **0.26** |  | **0.20** | 0.00 | 0.00 | 0.01 | 0.05 | | | -0.01 | | | -0.09 | |  |  |
|  | **(0.000)** | x | **(0.000)** | (0.976) | (0.990) | (0.976) | (0.405) | | | (0.953) | | | (0.084) | |  |  |
| **α-syn neurites** | **0.33** | **0.20** |  | 0.12 | 0.12 | **0.16** | -0.04 | | | -0.03 | | | **0.18** | |  |  |
|  | **(0.000)** | **(0.000)** | x | (0.077) | (0.084) | **(0.015)** | (0.651) | | | (0.755) | | | **(0.000)** | |  |  |
| **Aβ diffuse plaques** | 0.05 | 0.00 | 0.12 |  | **0.49** | **0.35** | 0.02 | | | 0.07 | | | **0.11** | |  |  |
|  | (0.438) | (0.976) | (0.077) | x | **(0.000)** | **(0.000)** | (0.782) | | | (0.298) | | | **(0.015)** | |  |  |
| **Aβ compact plaques** | 0.03 | 0.00 | 0.12 | **0.49** |  | 0.11 | -0.07 | | | **-0.20** | | | 0.05 | |  |  |
|  | (0.651) | (0.990) | (0.084) | **(0.000)** | x | (0.117) | (0.324) | | | **(0.000)** | | | (0.332) | |  |  |
| **Aβ classic-cored plaques** | 0.07 | 0.01 | **0.16** | **0.35** | 0.11 |  | -0.10 | | | -0.08 | | | **0.10** | |  |  |
|  | (0.298) | (0.976) | **(0.015)** | **(0.000)** | (0.117) | x | (0.077) | | | (0.208) | | | **(0.017)** | |  |  |
| **p-tau pretangles** | -0.03 | 0.05 | -0.04 | 0.02 | -0.07 | -0.10 |  | | | **0.50** | | | **0.16** | |  |  |
|  | (0.739) | (0.405) | (0.651) | (0.782) | (0.324) | (0.077) | x | | | **(0.000)** | | | **(0.000)** | |  |  |
| **p-tau NFTs** | -0.02 | -0.01 | -0.03 | 0.07 | **-0.20** | -0.08 | **0.50** | | |  | | | **0.21** | |  |  |
|  | (0.764) | (0.953) | (0.755) | (0.298) | **(0.000)** | (0.208) | **(0.000)** | | | x | | | **(0.000)** | |  |  |
| **p-tau threads** | 0.08 | -0.09 | **0.18** | **0.11** | 0.05 | **0.10** | **0.16** | | | **0.21** | | |  | |  |  |
|  | (0.129) | (0.084) | **(0.000)** | **(0.015)** | (0.332) | **(0.017)** | **(0.000)** | | | **(0.000)** | | | x | |  |  |
| **b.** |  |  |  |  |  |  |  | |  | |  | | |  | | |
| **vs.** | **Braak α-syn stage** | **MSA SND stage** | **MSA OPCA stage** | **Thal Aβ phase** | **Braak NFT stage** | **CAA stage** | **CERAD stage** | **TDP-LATE  stage** | | | | | | | |  |
| **α-syn GCIs** | 0.11 | 0.08 | 0.11 | 0.01 | -0.21 | -0.08 | -0.02 | 0.04 | | | |  | | | |  |
|  | (0.334) | (0.505) | (0.297) | (0.948) | (0.140) | (0.620) | (0.912) | (0.807) | | | |  | | | |  |
| **α-syn neuronal inclusions** | 0.14 | 0.11 | 0.07 | -0.04 | -0.16 | -0.04 | 0.04 | 0.06 | | | |  | | | |  |
|  | (0.212) | (0.358) | (0.577) | (0.807) | (0.286) | (0.807) | (0.815) | (0.656) | | | |  | | | |  |
| **α-syn neurites** | 0.22 | 0.09 | 0.08 | -0.02 | -0.20 | 0.07 | 0.10 | 0.17 | | | |  | | | |  |
|  | (0.052) | (0.515) | (0.594) | (0.912) | (0.227) | (0.691) | (0.594) | (0.209) | | | |  | | | |  |
| **Aβ diffuse plaques** | -0.01 | -0.11 | 0.03 | **0.34** | **0.27** | **0.24** | **0.28** | 0.09 | | | |  | | | |  |
|  | (0.912) | (0.212) | (0.807) | **(0.000)** | **(0.009)** | **(0.009)** | **(0.000)** | (0.452) | | | |  | | | |  |
| **Aβ compact plaques** | 0.01 | -0.16 | 0.04 | **0.31** | 0.26 | 0.16 | 0.09 | 0.14 | | | |  | | | |  |
|  | (0.925) | (0.183) | (0.807) | **(0.006)** | (0.062) | (0.241) | (0.209) | (0.359) | | | |  | | | |  |
| **Aβ classic-cored plaques** | 0.04 | 0.08 | -0.05 | **0.25** | 0.06 | 0.15 | **0.35** | **0.14** | | | |  | | | |  |
|  | (0.719) | (0.340) | (0.594) | **(0.001)** | (0.620) | (0.094) | **(0.000)** | **(0.018)** | | | |  | | | |  |
| **p-tau pretangles** | 0.06 | 0.01 | 0.00 | 0.13 | **0.33** | 0.14 | **0.27** | **0.22** | | | |  | | | |  |
|  | (0.594) | (0.925) | (0.948) | (0.209) | **(0.001)** | (0.195) | **(0.009)** | **(0.009)** | | | |  | | | |  |
| **p-tau NFTs** | 0.03 | -0.02 | 0.03 | **0.23** | **0.28** | 0.16 | **0.23** | **0.19** | | | |  | | | |  |
|  | (0.785) | (0.887) | (0.785) | **(0.009)** | **(0.009)** | (0.140) | **(0.009)** | **(0.036)** | | | |  | | | |  |
| **p-tau threads** | -0.02 | -0.06 | -0.06 | **0.24** | **0.54** | 0.08 | **0.29** | 0.10 | | | |  | | | |  |
|  | (0.860) | (0.620) | (0.656) | **(0.029)** | **(0.000)** | (0.616) | **(0.014)** | (0.405) | | | |  | | | |  |
| **c.** |  |  |  |  |  |  |  | |  | |  | | |  | | |
| **vs.** | **Age at onset** | **Age at death** | **Misdiagnoses** | **Disease duration** | **CDR** | **APOE-ε4** | **Hallucinations** | |  | |  | | |  | | |
| **α-syn GCIs** | **-0.88** | **-0.20** | 0.00 | **0.30** | 0.10 | 0.01 | 0.06 | |  | |  | | |  | | |
|  | **(0.000)** | **(0.022)** | (0.970) | **(0.000)** | (0.344) | (0.933) | (0.677) | |  | |  | | |  | | |
| **α-syn neuronal inclusions** | **-0.91** | -0.12 | 0.02 | **0.32** | 0.15 | 0.05 | -0.003 | |  | |  | | |  | | |
|  | **(0.000)** | (0.216) | (0.813) | **(0.000)** | (0.157) | (0.644) | (0.970) | |  | |  | | |  | | |
| **α-syn neurites** | **-0.70** | -0.08 | 0.11 | **0.26** | **0.24** | 0.03 | -0.09 | |  | |  | | |  | | |
|  | **(0.021)** | (0.470) | (0.344) | **(0.007)** | **(0.027)** | (0.778) | (0.525) | |  | |  | | |  | | |
| **Aβ diffuse plaques** | 0.30 | **0.25** | 0.16 | -0.10 | **0.24** | **0.22** | -0.08 | |  | |  | | |  | | |
|  | (0.216) | **(0.001)** | (0.050) | (0.213) | **(0.001)** | **(0.002)** | (0.456) | |  | |  | | |  | | |
| **Aβ compact plaques** | 0.34 | **0.23** | **0.21** | -0.13 | **0.25** | 0.11 | -0.04 | |  | |  | | |  | | |
|  | (0.263) | **(0.020)** | **(0.040)** | (0.214) | **(0.015)** | (0.332) | (0.480) | |  | |  | | |  | | |
| **Aβ classic-cored plaques** | 0.14 | **0.24** | 0.11 | -0.03 | 0.03 | **0.23** | -0.04 | |  | |  | | |  | | |
|  | (0.509) | **(0.001)** | (0.146) | (0.644) | (0.773) | **(0.001)** | (0.450) | |  | |  | | |  | | |
| **p-tau pretangles** | 0.06 | **0.38** | **0.21** | -0.03 | 0.03 | 0.08 | 0.09 | |  | |  | | |  | | |
|  | (0.797) | **(0.000)** | **(0.005)** | (0.744) | (0.778) | (0.344) | (0.480) | |  | |  | | |  | | |
| **p-tau NFTs** | 0.18 | **0.33** | **0.18** | -0.07 | 0.08 | 0.11 | 0.03 | |  | |  | | |  | | |
|  | (0.451) | **(0.000)** | **(0.020)** | (0.370) | (0.385) | (0.216) | (0.537) | |  | |  | | |  | | |
| **p-tau threads** | 0.28 | **0.50** | 0.18 | -0.08 | 0.12 | 0.16 | -0.13 | |  | |  | | |  | | |
|  | (0.344) | **(0.000)** | (0.062) | (0.432) | (0.242) | (0.110) | (0.490) | |  | |  | | |  | | |
| **d.** |  |  |  |  |  |  |  | |  | |  | | |  | | |
| **vs.** | **Age at onset** | **Age at death** | **Misdiagnoses** | **Disease duration** | **CDR** | **APOE-ε4** |  | |  | |  | | |  | | |
| **Braak α-syn stage** | 0.04 | -0.07 | 0.08 | 0.10 | 0.02 | 0.18 |  | |  | |  | | |  | | |
|  | (0.853) | (0.806) | (0.920) | (0.781) | (0.964) | (0.440) |  | |  | |  | | |  | | |
| **MSA SND stage** | 0.10 | 0.06 | -0.66 | -0.07 | 0.07 | 0.00 |  | |  | |  | | |  | | |
|  | (0.781) | (0.806) | (0.249) | (0.806) | (0.806) | (0.995) |  | |  | |  | | |  | | |
| **MSA OPCA stage** | -0.09 | -0.16 | 0.24 | 0.05 | -0.29 | -0.06 |  | |  | |  | | |  | | |
|  | (0.806) | (0.442) | (0.806) | (0.841) | (0.080) | (0.806) |  | |  | |  | | |  | | |
| **Thal Aβ phase** | **0.44** | **0.48** | 0.24 | **0.38** | 0.15 | 0.03 |  | |  | |  | | |  | | |
|  | **(0.000)** | **(0.000)** | (0.806) | **(0.002)** | (0.442) | (0.917) |  | |  | |  | | |  | | |
| **Braak NFT stage** | **0.30** | **0.59** | 0.30 | **0.35** | 0.03 | -0.09 |  | |  | |  | | |  | | |
|  | **(0.009)** | **(0.000)** | (0.690) | **(0.001)** | (0.917) | (0.806) |  | |  | |  | | |  | | |
| **CAA stage** | **0.47** | **0.39** | 0.22 | **0.31** | 0.18 | -0.06 |  | |  | |  | | |  | | |
|  | **(0.000)** | **(0.005)** | (0.806) | **(0.029)** | (0.411) | (0.806) |  | |  | |  | | |  | | |
| **CERAD stage** | **0.58** | **0.38** | 0.05 | **0.62** | 0.12 | -0.01 |  | |  | |  | | |  | | |
|  | **(0.000)** | **(0.011)** | (0.964) | **(0.000)** | (0.704) | (0.995) |  | |  | |  | | |  | | |
| **TDP-LATE stage** | 0.00 | **0.34** | 0.48 | -0.07 | 0.18 | 0.23 |  | |  | |  | | |  | | |
|  | (0.995) | **(0.033)** | (0.442) | (0.806) | (0.440) | (0.207) |  | |  | |  | | |  | | |

**Associations between neuropathological burden, pathological staging and clinical variables across all regions.** Standardized regression coefficients (r) calculated from the β-value in a mixed-effects linear regression, corrected for age and sex; r-value and *p*-value (between brackets) are depicted below each other. Significant *p*-values are demonstrated in **bold**

**
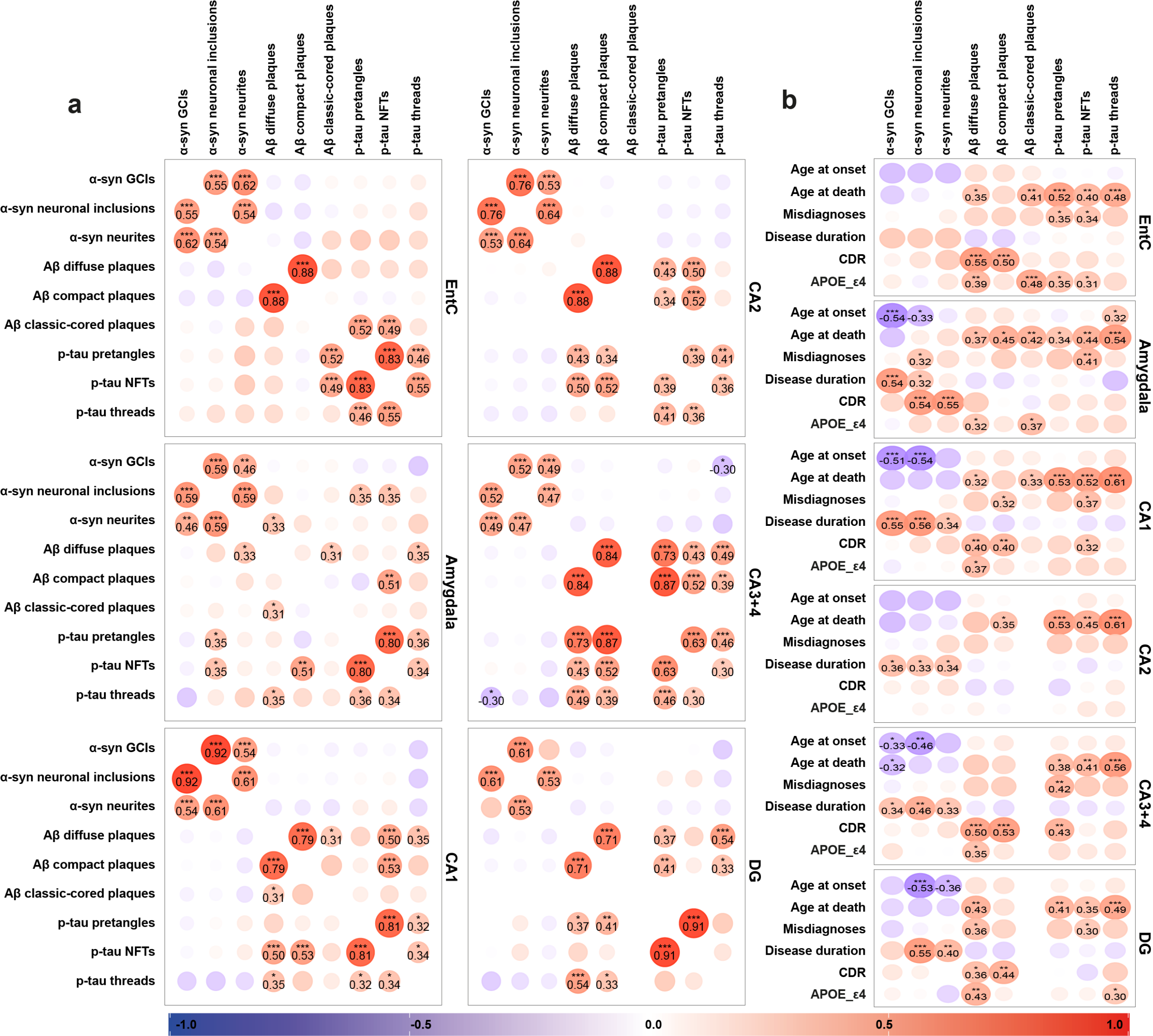
11. Regional vulnerability in neuropathological and clinicopathological correlations.** Bubble plots show partial correlations (r) across brain regions (EntC, amygdala, CA1, CA2, CA3+4 and DG). **a** Pairwise correlations between α-syn, Aβ and p-Tau pathological burden. **b** Correlations between standardized clinical variables (rows) and neuropathological burden (columns). Correlations were derived as Pearson correlations between residuals from linear models adjusting for age at death and sex, and represent partial associations. Bubble size represents the absolute strength of the correlation, color indicates the direction (red = positive, blue = negative). Significance after FDR-correction is demonstrated in bold as * p < 0.05, ** p < 0.01, and *** p < 0.001

**References**

1. Braak H, Del Tredici K, Rüb U, de Vos RA, Jansen Steur EN, Braak E. Staging of brain pathology related to sporadic Parkinson's disease. Neurobiol Aging. 2003;24(2):197-211.

2. Braak H, Alafuzoff I, Arzberger T, Kretzschmar H, Del Tredici K. Staging of Alzheimer disease-associated neurofibrillary pathology using paraffin sections and immunocytochemistry. Acta Neuropathol. 2006;112(4):389-404.

3. Thal DR, Rüb U, Orantes M, Braak H. Phases of A beta-deposition in the human brain and its relevance for the development of AD. Neurology. 2002;58(12):1791-800.

4. Jellinger KA, Seppi K, Wenning GK. Grading of neuropathology in multiple system atrophy: proposal for a novel scale. Mov Disord. 2005;20 Suppl 12:S29-36.

5. Nelson PT, Lee EB, Cykowski MD, Alafuzoff I, Arfanakis K, Attems J, et al. LATE-NC staging in routine neuropathologic diagnosis: an update. Acta Neuropathol. 2023;145(2):159-73.

6. Thal DR, Griffin WS, de Vos RA, Ghebremedhin E. Cerebral amyloid angiopathy and its relationship to Alzheimer's disease. Acta Neuropathol. 2008;115(6):599-609.

7. Mirra SS, Heyman A, McKeel D, Sumi SM, Crain BJ, Brownlee LM, et al. The Consortium to Establish a Registry for Alzheimer's Disease (CERAD). Part II. Standardization of the neuropathologic assessment of Alzheimer's disease. Neurology. 1991;41(4):479-86.

8. Kovacs GG, Ferrer I, Grinberg LT, Alafuzoff I, Attems J, Budka H, et al. Aging-related tau astrogliopathy (ARTAG): harmonized evaluation strategy. Acta Neuropathol. 2016;131(1):87-102.

9. Braak H, Braak E. Cortical and subcortical argyrophilic grains characterize a disease associated with adult onset dementia. Neuropathol Appl Neurobiol. 1989;15(1):13-26.

10. van Wetering J, Geut H, Bol JJ, Galis Y, Timmermans E, Twisk JWR, et al. Neuroinflammation is associated with Alzheimer's disease co-pathology in dementia with Lewy bodies. Acta Neuropathol Commun. 2024;12(1):73.

11. Thal DR, Ghebremedhin E, Rüb U, Yamaguchi H, Del Tredici K, Braak H. Two types of sporadic cerebral amyloid angiopathy. J Neuropathol Exp Neurol. 2002;61(3):282-93.
